# Coding and non-coding variants in the ciliopathy gene *CFAP410* cause early-onset non-syndromic retinal degeneration

**DOI:** 10.1038/s41525-024-00439-3

**Published:** 2024-11-08

**Authors:** Riccardo Sangermano, Priya Gupta, Cherrell Price, Jinu Han, Julien Navarro, Christel Condroyer, Emily M. Place, Aline Antonio, Shizuo Mukai, Xavier Zanlonghi, José-Alain Sahel, Stephanie DiTroia, Emily O’Heir, Jacque L. Duncan, Eric A. Pierce, Christina Zeitz, Isabelle Audo, Rachel M. Huckfeldt, Kinga M. Bujakowska

**Affiliations:** 1 Ocular Genomics Institute, Department of Ophthalmology, Massachusetts Eye and Ear Infirmary, Harvard Medical School, Boston, MA USA; 2 Department of Ophthalmology, Gangnam Severance Hospital, Institute of Vision Research, Yonsei University College of Medicine, Seoul, Republic of Korea; 3 Sorbonne Université, INSERM, CNRS, Institut de la Vision, Paris, France; 4 Retina Service, Department of Ophthalmology, Massachusetts Eye and Ear, Harvard Medical School, Boston, MA USA; 5 Centre de compétence maladies rares, Service d’Ophtalmologie, CHU Rennes, Rennes, France; 6 Centre Hospitalier National d’Ophtalmologie des Quinze-Vingts, Centre de Référence Maladies Rares REFERET and INSERM-DGOS, CIC 1423 Paris, France; 7 Vision Institute, University of Pittsburgh Medical Center and School of Medicine, Pittsburgh, PA USA; 8 Center for Mendelian Genomics, Broad Institute of Massachusetts Institute of Technology and Harvard, Cambridge, MA USA; 9 Department of Ophthalmology, University of California, San Francisco, CA USA

**Keywords:** Mutation, Genetic counselling, Genetic association study, Next-generation sequencing

## Abstract

Inherited retinal degenerations are blinding genetic disorders characterized by high genetic and phenotypic heterogeneity. In this retrospective study, we describe sixteen families with early-onset non-syndromic retinal degenerations in which affected probands carried rare bi-allelic variants in *CFAP410*, a ciliary gene previously associated with recessive Jeune syndrome. We detected twelve variants, eight of which were novel, including c.373+91A>G, which led to aberrant splicing. To our knowledge this is the first likely pathogenic deep-intronic variant identified in this gene. Analysis of all reported and novel *CFAP410* variants revealed no clear correlation between the severity of the *CFAP410-*associated phenotypes and the identified causal variants. This is supported by the fact that the frequently encountered missense variant p.(Arg73Pro), often found in syndromic cases, was also associated with non-syndromic retinal degeneration. This study expands the current knowledge of *CFAP410*-associated ciliopathy by enriching its mutational landscape and supports its association with non-syndromic retinal degeneration.

## Introduction

Inherited retinal degenerations (IRDs) are a group of genetically and clinically heterogeneous disorders characterized by progressive loss of cone and rod photoreceptors. IRDs can manifest as an isolated phenotype, where only retina is affected (i.e., non-syndromic IRDs) or as a syndromic disease, where retinal degeneration is one of many signs of a multiorgan clinical manifestation. IRDs can be classified based on their onset (early *vs* late) and/or photoreceptor degeneration patterns (cone dystrophy (CD), cone-rod dystrophy (CRD), and rod-cone dystrophy (RCD)^1^.

The Cilia and Flagella Associated Protein 410 *(CFAP410)* gene (OMIM 603191), formerly known as *C21orf2*, is a ciliary gene of unclear specific function. Given its mapping position on chromosome 21, *CFAP410* was initially thought to play a role in the pathogenesis of several genetic diseases including Trisomy 21 (Down syndrome), but none of these associations have been confirmed^2–4^.

Functional genomic screens for ciliary gene identification^5,6^ combined with mutational screening in unsolved ciliopathy patients confirmed the essential role of the CFAP410 protein in ciliogenesis. Individuals with bi-allelic pathogenic variants in this gene were reported to have Jeune syndrome (JS)^6^, a recessive skeletal ciliopathy (OMIM# 611263)^7,8^ also known as asphyxiating thoracic dystrophy and axial spondylometaphyseal dysplasia (SMDAX)^9^. Affected individuals usually present with shortened ribs and a narrowed chest accompanied by other skeletal abnormalities, but retinal degeneration and other non-skeletal features can be also present^8^.

Many ciliopathy cases harboring pathogenic *CFAP410* variants have been described to date^6,9–17^. However, in 2015, Khan and colleagues described a specific phenotype of early-onset retinal dystrophy with macular staphyloma but without high myopia in three Saudi families with a history of consanguinity and carrying homozygous variants in *CFAP410*^18^. Since then, a few other non-syndromic *CFAP410* cases have been reported as a consequence of mutational screens in large IRD cohorts^10–13,19–35^. However, a conclusive association of *CFAP410* mutations with non-syndromic IRD has never been reached due to the small number of non-syndromic cases. In this study, we describe fourteen new families with early-onset non-syndromic retinal degeneration and two additional cases with a milder form of JS that confirm the phenotype expansion for bi-allelic variants in *CFAP410*. We also report eight novel variants in this gene, six of which are pathogenic or likely pathogenic.

## Results

### Clinical phenotypes

**Fig. 1:**
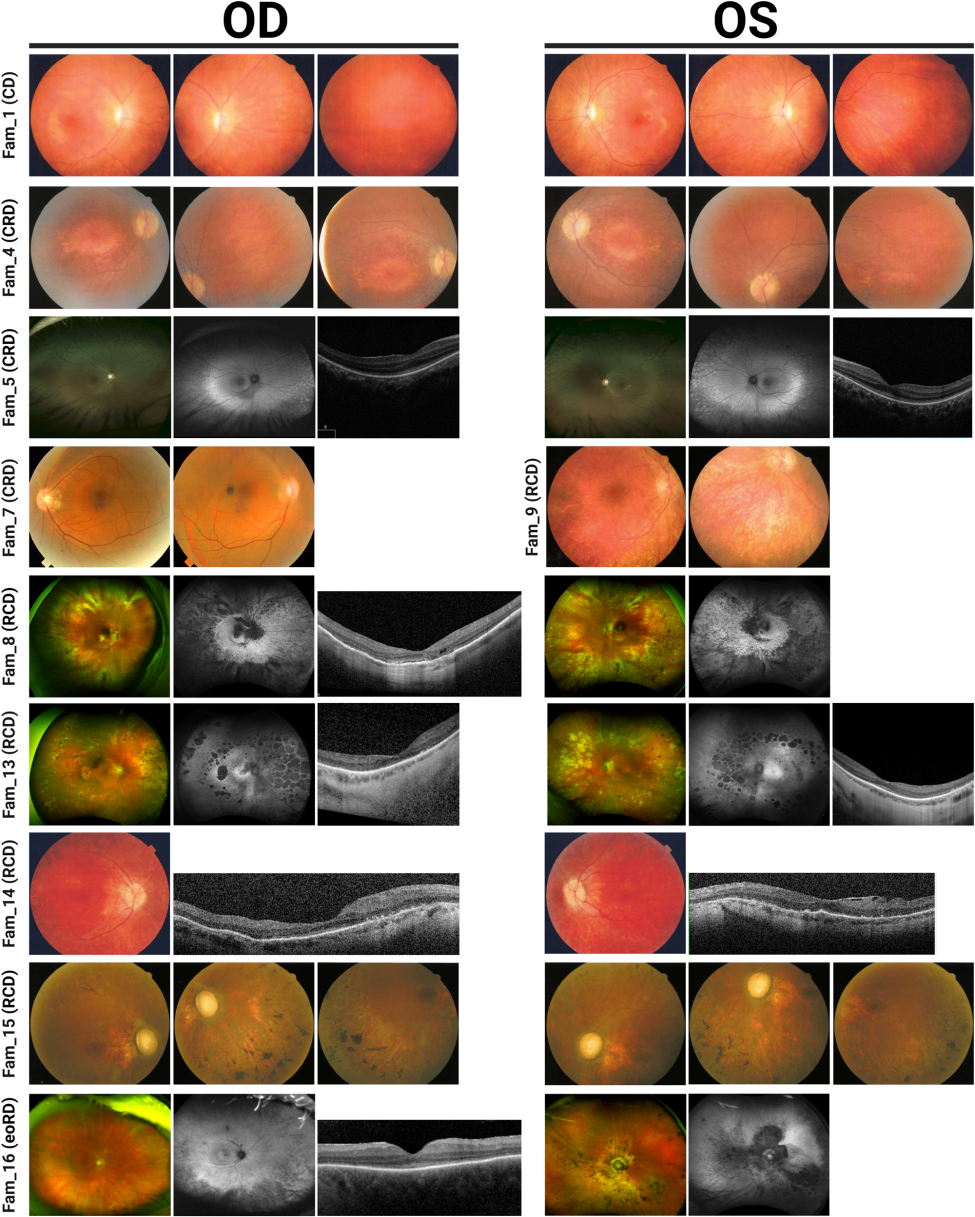
Clinical phenotypes of *CFAP410*-IRD patients. Images show fundus photos for a representative subset of individuals. Fundus autofluorescence and/or OCT imaging were available for five individuals (5, 8, 13, 14, and 16) and showed features consistent with the fundus findings and clinical diagnosis. The specific IRD phenotype of each patient is given in brackets (CD cone dystrophy, CRD cone-rod dystrophy, RCD rod-cone dystrophy, eoRD early-onset retinal dystrophy). Note the tapetal-like sheen in fundus images in proband 5 with CRD, and the morning glory disc in the left eye of proband 16 with eoRD.

Sixteen probands (seven females and nine males) with *CFAP410*-associated disease had clinical phenotypes falling into four diagnostic categories: early-onset retinal dystrophy (eoRD; *n* = 1), cone dystrophy (CD; *n* = 1), cone-rod dystrophy (CRD; *n* = 6), and rod-cone dystrophy (RCD; *n* = 8) (see Fig. 1, Table 1, and Supplementary Table 1 for detailed clinical data). In most cases, the symptom onset occurred in childhood, prior to the age of 10, and at the initial clinical evaluation, the individuals were 9–71 years of age. The presenting symptom typically corresponded to the clinical diagnosis (for example, nyctalopia in RCD).

**Table 1:** Clinical characteristics of IRD probands carrying *CFAP410* variants

Family_ Proband	Genotype^a^	Proband_ Research_ID	Age at initial visit; Gender	Ethnicity	Dx	Non-retinal features	Ocular symptoms
1_II-1	V1|V1	OGI3083_ 4678	13|M	White (Scottish/ Irish/Italian)	CD	None^d^	Light sensitivity, ↓ central vision
2_II-1	V1|V1	OGI3014_ 4600	25|F	White^b^ (Irish)	CRD	None^d^	Poor vision since age 6; photosensitivity
3_II-1	V2|V3	OGI1446_ 2641	32|M	White (Italian)	CRD	Premature birth, no skeletal abnormalities reported or seen on extensive x-rays	↓ central vision at age 6, ↓ color vision at age 18, ↓ night vision at age 19
4_II-1	V1|V4	OGI3006_ 4592	22|F	n.a.	CRD	None^d^	↓ central vision since childhood; ↓ peripheral vision in teens
5_II-1	V5|V6	OGI3900_ 53031	9|M	Japanese	CRD	None^d^	↓ vision at age 1
6_II-1	V7|V7	CIC07923_F4428	51|M	Black (Senegalese)	CRD	None^d^	Diagnosis in childhood of nyctalopia and severely decreased VA
7_II-1	V8|V9	OGI3057_ 4647	45|M	Indian	CRD	None^d^	↓ central vision since age 18 and night vision since age 30, delayed dark adaptation
8_II-1	V1|V10	OGI2139_ 3573	38|F	White (English/Irish)	RCD	None^d^	↓ central vision since age 7; ↓ night and peripheral vision since age 28
9_II-1	V1|V1	OGI1877_ 3254	12|F	White (Irish)	RCD	None^d^	Nyctalopia and ↓ peripheral vision since age 4
10_II-1	V1|V1	CIC04687_F2325	38|M	White (Breton)	RCD	None^d^	Nyctalopia since early childhood, never had VA above 20/32, progressive visual field constriction, diagnosis of RCD at 14
11_II-1	V1|V1	CIC03728_F1669	63|F	White (Breton)	RCD	None^d^	Nyctalopia since teens, progressive visual field constriction, diagnosis of RCD in her 20s
12_II-1	V1|V1	CIC01570_F4189	71|F	White (Breton)	RCD	None^d^	Diagnosis in childhood of nyctalopia and progressive field constriction
13_II-1	V1|V1	OGI1309_ 2454	57|M	White	RCD	None^d^	Nyctalopia and ↓ peripheral vision since childhood
14_II-1	V1|V1	OGI1369_ 2543	32|M	White (Scottish)	RCD	Thoracic skeletal abnormalities^e^	Nyctalopia since childhood
15_II-1	V11|V11	OGI2251_ 3735	46|M	Black (Haitian^c^)	RCD	None^d^	Nyctalopia since age 4, ↓ peripheral vision since age 25 and central vision since age 36
16_II-1	V12|V12	OGI3844_ 52295	50|F	White	eoRD	Bilateral hip dysplasia, asymmetric bilateral hearing loss (40 s), early ovarian failure (30)	Nystagmus as an infant. Nyctalopia since early childhood. Never 20/20; ↓ central vision in 20s. ↓ peripheral vision in 40s

*CD* cone dystrophy, *CRD* cone-rod dystrophy, *Dx* diagnosis (supported by ERG findings, see Supplementary Table 1), *eoRD* early-onset retinal dystrophy, *F* female, *M* male, *n.a.* not available, *RCD* rod-cone dystrophy.

**Fig. 2:**
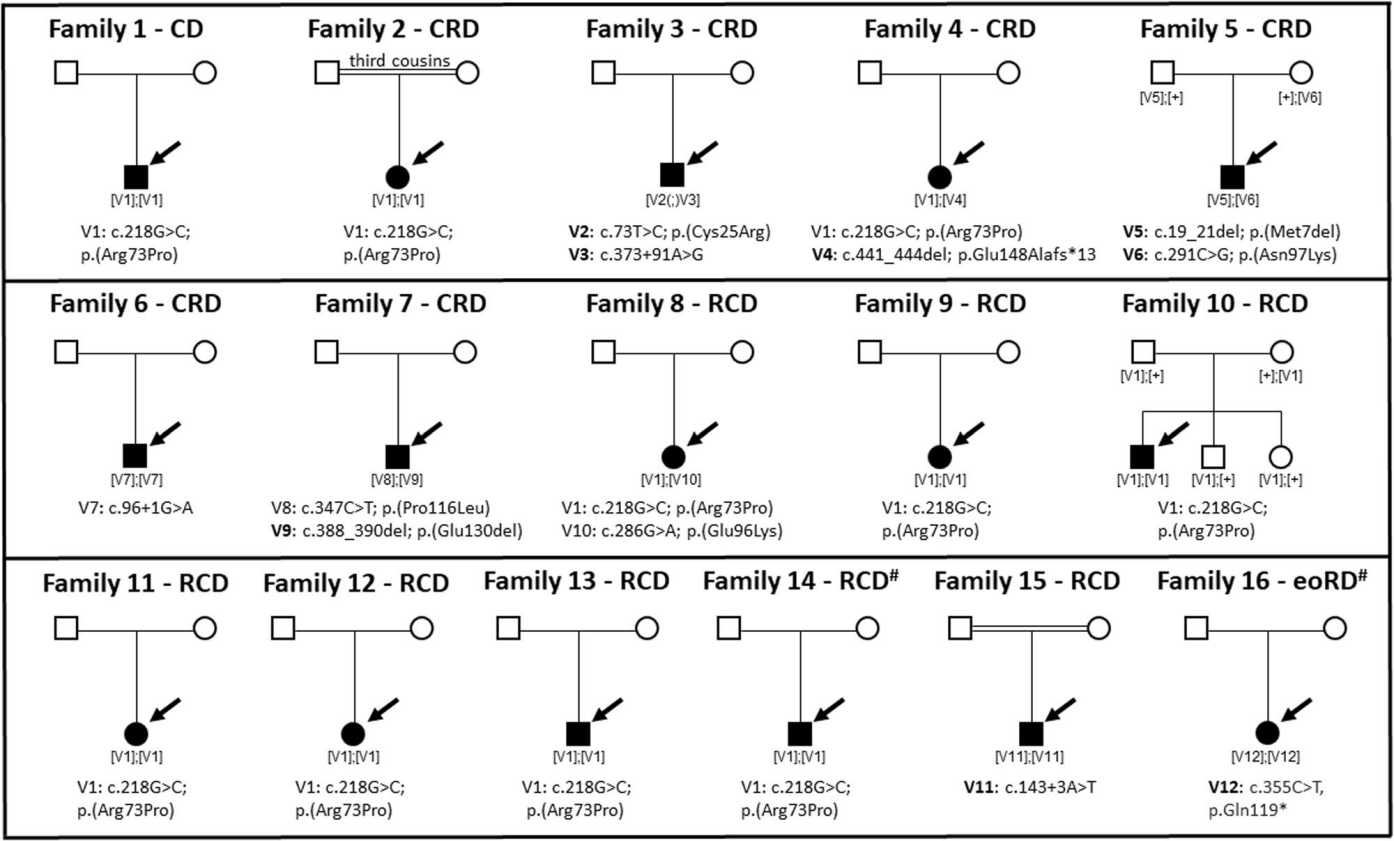
Pedigrees of the 16 *CFAP410* families described in this study. For each family (1–16), the specific IRD phenotype diagnosed is mentioned above each pedigree (CD cone dystrophy, CRD cone-rod dystrophy, RCD rod-cone dystrophy, eoRD early-onset retinal dystrophy). Mildly syndromic families 14 and 16 are indicated with a hashtag (#). Affected male and female subjects are represented with black squares or circles, respectively. Probands are indicated by a black arrow. Novel variants are indicated in bold. First cousin marriage is indicated by a double-line. All presented variants refer to the *CFAP410* transcript NM_004928.3. Bi-allelic inheritance was confirmed by familial segregation analysis (families 5 and 10), by ruling out deletion events in *CFAP410* bioinformatically (families 1, 2, 6, 9, 11, 12, 13, 14, 15, 16), by analysis of NGS pair-end reads (family 8), and by cloning and by using the gnomAD v2 Variant Co-Occurrence tool (families 4 and 7). In family 3 we could not confirm bi-allelic inheritance, thus variants are indicated as [V(;)V].

^a^See Fig. 2 and Table 2 for variant details.

^b^Parents are third cousins.

^c^Parents are first cousins.

^d^No skeletal abnormalities on questionnaire.

^e^Prior rib cage procedure.

Visual acuity was significantly reduced at young ages regardless of clinical diagnosis. The youngest proband with CRD (proband 5) had visual acuity of 20/100 and 20/125 when evaluated at age 9, and the youngest proband with RCD (proband 9) had visual acuity of 20/100 in each eye at age 12. Except probands of families 10 and 11, no individual in the cohort had visual acuity better than 20/80 (see Supplementary Table 1), and fourteen eyes of eight individuals had visual acuity at or beyond the threshold of legal blindness at the initial evaluation.

When available, visual field data from Goldmann kinetic perimetry showed better overall preservation of visual fields in patients with clinical diagnoses of CD/CRD whereas most with RCD had constriction sparing only the central visual fields. Full-field ERGs were available for all patients. Individuals with clinical diagnoses of CD and CRD showed varying degrees of scotopic compromise with more severe photopic dysfunction; the scotopic responses for proband 2 did show deterioration over two studies spanning 12 years. Individuals with RCD had severe generalized dysfunction of scotopic and photopic responses.

Fundus evaluation showed features that were typical for the retinal diagnosis (Fig. 1). Staphylomas were noted in two individuals (probands 4, 8). Digital OCT images were available for eight individuals and showed significant attenuation and absence of photoreceptor bands, particularly in the peripheral macula with relatively better preservation of foveal lamination. Visual acuity was lower than might be expected from the remaining structure with the structure vs. function dissociations in probands 5, 13 (OS), and 16 particularly illustrative of this observation. OCT suggested posterior staphyloma in one individual for whom it was not noted on clinical exam (proband 13).

Additional ophthalmic diagnoses included amblyopia (proband 13), bilateral pseudophakia (probands 11 and 12), history of strabismus surgery (proband 14), and nystagmus (probands 2, 16).

Skeletal abnormalities were present in two individuals: proband 14 had thoracic skeletal abnormalities requiring surgical intervention, and proband 16 had bilateral hip dysplasia corrected with hip replacement. No other individuals had skeletal abnormalities present on imaging (proband 3) or self-report. Proband 16 also had premature ovarian failure at age 30 as well as bilateral sensorineural hearing loss beginning in her 40 s, but no other systemic diagnoses of note were present in the cohort.

### Rare *CFAP410* variants associated with non-syndromic early-onset IRD

By analyzing data from either targeted next generation sequencing, exome sequencing (ES), or genome sequencing (GS) of a cohort of 7000 IRD cases, we identified 12 rare *CFAP410* variants (V1-12, MAF < 0.0006) in 16 probands and their family members (see Fig. 2 and Table 2). No additional disease-causing variants were present in any of the currently known IRD genes^36^ that were able to explain the clinical phenotype.

**Table 2:** The twelve *CFAP410* variants identified in our patients

*CFAP410* variant	GnomAD v4_AF	Reported in LOVD	Reported in our study	ACMG classification	ACMG criteria	Associated phenotype(s)	References
**V1**: c.218G>C; p.(Arg73Pro)	0.0005023	26	10	LP	PM1; PM2; PM3; PP4	CD; CRD; RCD; RD; SMDAX + RCD; JS + CRD; JS + RD^a^	Wheway et al. ^6^; Wang et al. ^9^; Zhang et al. ^31^ Carss et al. ^32^; McInerney-Leo et al. ^14^; Lionel et al. ^33^; Holtan et al. ^22^; Rodriguez-Munoz et al. ^20^; Weisschuh et al. ^24^; Turro et al. ^37^; Fadaie et al. ^25^; Tracewska et al. ^29^; Weisschuh et al. ^27^; Hitti-Malin et al. ^26^; this study
**V2**: c.73T>C; p.(Cys25Arg)	6.924e-7	0	1 (novel)	LP	PM1; PM2; PP3; PP4	CRD	this study
**V3**: c.373+91A>G	n.a.	0	1 (novel)	LP	PS3; PM2; PP4	CRD	this study
**V4**: c.441_444del; p.Glu148Alafs*13	0.000005579	0	1 (novel)	P	PVS1; PM2; PM3; PP4; PP5	CRD	this study
**V5**: c.19_21del; p.(Met7del)	6.799e-7	0	1 (novel)	VUS	PM2; PM4; PP4	CRD	this study
**V6**: c.291C>G; p.(Asn97Lys)	0.000001241	0	1 (novel)	VUS	PM2; PP4	CRD	this study
**V7**: c.96+1G>A	0.000001240	3	1	P	PVS1; PM2; PP3; PP4; PP5	CRD	Huang et al. ^30^; Rodriguez-Munoz et al. ^20^; this study
**V8**: c.347C>T; p.(Pro116Leu)	0.00002813	3	1	LP	PM1; PM2; PM3; PP3; PP4	CRD; SMDAX	Wang et al. ^9^; Villafuerte-de la Cruz et al. ^28^; this study
**V9**: c.388_390del; p.(Glu130del)	0.000002495	0	1 (novel)	LP	PM1; PM2; PM3; PP4	CRD	this study
**V10**: c.286G>A; p.(Glu96Lys)	0.000006829	2	1	LP	PM2; PM3; PP3; PP4	CRD; RCD; SMDAX + CRD	De Castro-Miró et al. ^11^; Weisschuh et al. ^24^; this study
**V11**: c.143+3A>T	6.304e-7	0	1 (novel)	LP	PS3; PM2; PP3; PP4	RCD	this study
**V12**: c.355C>T; p.Gln119*	0.00002069	0	1 (novel)	P	PVS1; PM2; PP4; PP5	eoRD	this study

*CD* cone dystrophy, *CRD* cone-rod dystrophy, *eoRD* early-onset retinal dystrophy, *JS* Jeune Syndrome, *LP* likely pathogenic, *n.a.* not available, *P* pathogenic, *RCD* rod-cone dystrophy, *RD* retinal degeneration, *SMDAX* Axial spondylometaphyseal dysplasia, *VUS* variant of uncertain significance.

^a^See Supplementary Table 3 for the complete list of phenotypes associated with the p.(Arg73Pro) variant.

The coding variants detected were truncating (p.Gln119* and p.Glu148Alafs*13), missense (p.Cys25Arg, p.Arg73Pro, p.Glu96Lys, p.Asn97Lys, p.Pro116Leu), or leading to single amino acid deletions (p.Met7del and p.Glu130del), while the non-coding variants c.96+1G>A, c.143+3A>T and c.373+91A>G were located in *CFAP410* intron 2, 3 and 4, respectively.

Most of the detected variants were novel, except for c.96+1G>A^20,30^, p.Arg73Pro^6,9,14,20,22,24–27,29,31–33,37^, p.Glu96Lys^11,24^, and p.Pro116Leu^9,28^ which were reported in patients with syndromic and non-syndromic IRD (See Supplementary Tables 2 and 3). The p.Arg73Pro was the most commonly reported variant and also the most common in our cohort: present homozygously in eight probands and heterozygously in two (families 4 and 8, see Fig. 2). However, this variant remains extremely rare in the general population, given the allele frequency in Genome Aggregation Database (gnomAD) v4 of 0.0005023^38^. Consanguinity was reported only in families 2 (c.218G>C, p.Arg73Pro) and 15 (c.143+3A>T), in which the parents were third and first cousins, respectively. An additional proband 16 was homozygous for the c.355C>T, p.(Gln119*) variant, though no consanguinity was noted.

Bi-allelic inheritance in the homozygous cases was confirmed by familial segregation analysis (family 10) or by ruling out deletion events in *CFAP410* bioinformatically. Compound heterozygosity was confirmed by familial segregation analysis (family 5); analysis of NGS pair-end reads (family 8), by cloning and by using the gnomAD v2 Variant Co-Occurrence tool (https://gnomad.broadinstitute.org/variant-cooccurrence) (families 4 and 7) (see Supplementary Figs. 1 and 2). Unfortunately, we could not use these methods to validate the phase of the variants identified in family 3, the c.73T>C; p.(Cys25Arg) and the c.373+91A>G. Both alleles were absent from gnomAD v2 and they were too far apart (~6 kb) to be cloned in one single fragment, given the limited quality of the historical DNA samples available. Only variant c.73T>C; p.(Cys25Arg) was present in one individual in the recently released version of GnomAD v4, while variant c.373+91A>G was absent (see Table 2). However this data is too scarce to conclude definitively if these two variants are likely in *cis* or *trans*.

### Novel non-coding *CFAP410* variants lead to splicing defects

To investigate the effect of c.143+3A>T and c.373+91A>G on pre-mRNA splicing we generated wild-type and variant mini-gene splicing constructs, which were transfected into HEK293T cells. The effect on splicing was investigated by RT-PCR. Both variants were predicted to affect normal splicing according to multiple in silico tools, such as SpliceSiteFinder-like^39^, MaxEntScan^40^, NNSPLICE^41^, GeneSplicer^42^, Human Splicing Finder^43^, and SpliceAI^44^. Variant c.143+3A>T was predicted to disrupt the splice donor site of *CFAP410* exon 3, while c.373+91A>G was predicted to generate a strong splice acceptor site in intron 4 (see Supplementary Figs. 3 and 4).

**Fig. 3:**
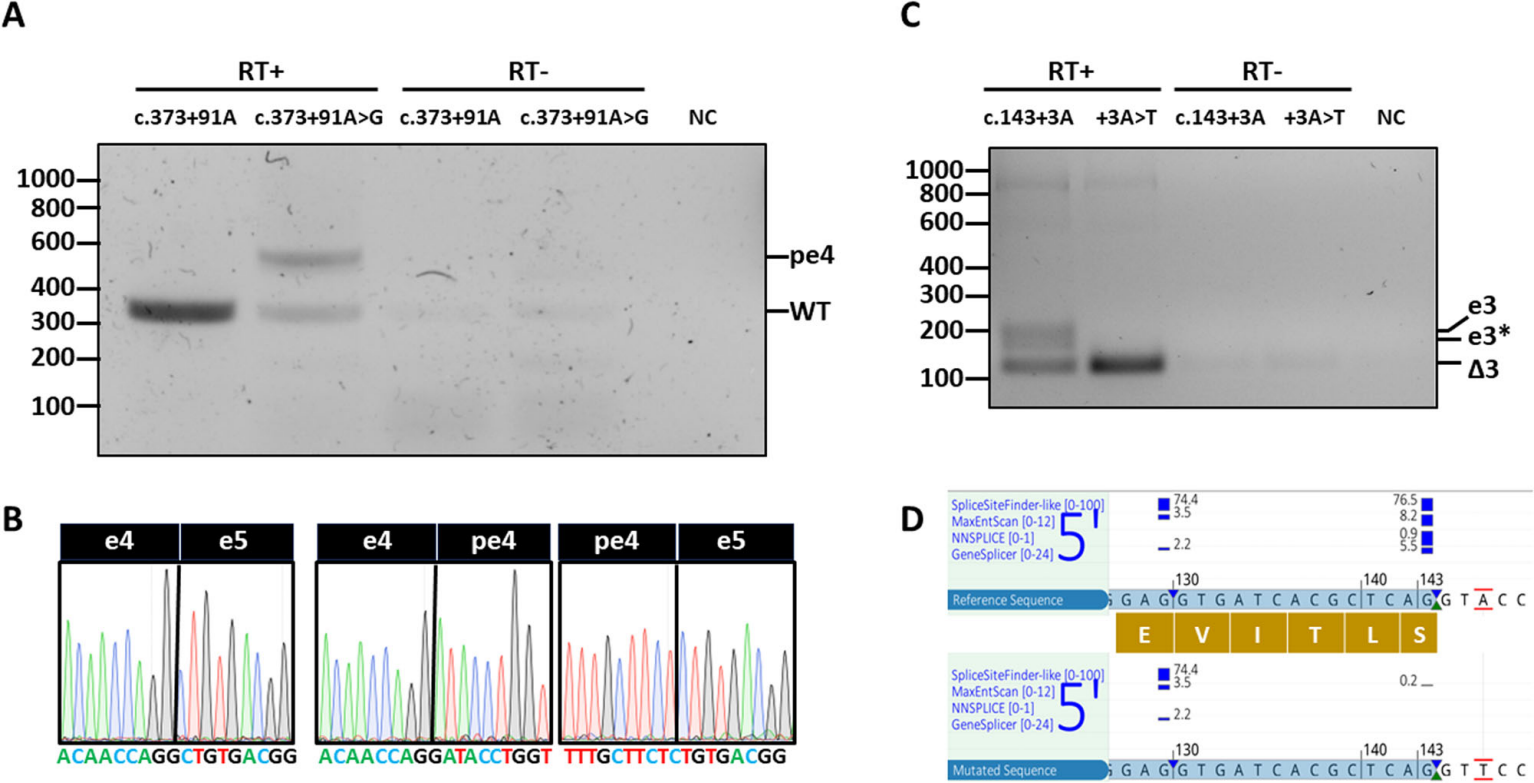
Functional validation of *CFAP410* splicing variants c.373+91A>G and c.143+3A>T. **A** RT-PCR showing the formation of a pseudoexon in intron 4 (pe4) in the construct containing the *CFAP410* c.373+91A>G variant compared to the wild-type (WT) band generated by the reference construct. RT-PCR reaction was performed using as input either retrotrascribed (RT+) or not retrotrascribed (RT-) RNA samples. NC, negative control. **B** Sanger sequencing of the splice boundaries between exon 4 and 5, confirming the breakpoint of the pseudoexon. **C** RT-PCR showing the skipping of exon 3 (Δ3) in the construct containing the c.143+3A>T compared to the wild-type (WT) construct, which generates both a full (e3) and truncated (e3*) version of exon 3, according to the splicing prediction **D**.

The splicing assay confirmed the presence of aberrant splicing phenotypes for both variants (see Fig. 3 and Supplementary Fig. 5). Indeed, exon 3 was skipped in the construct carrying the c.143+3A>T, while the splice acceptor created by c.373+91A>G resulted in the inclusion of a 200- base pair pseudoexon, previously predicted by SpliceAI (see Supplementary Fig. 4), in at least half of the transcripts according to our splicing assay (see Fig. 3A). Both splicing defects were classified as severe and fully penetrant, as they caused frameshift and premature stop codon in all generated transcripts (see Fig. 3).

### Protein modeling and genotype-phenotype correlation analysis

**Fig. 4:**
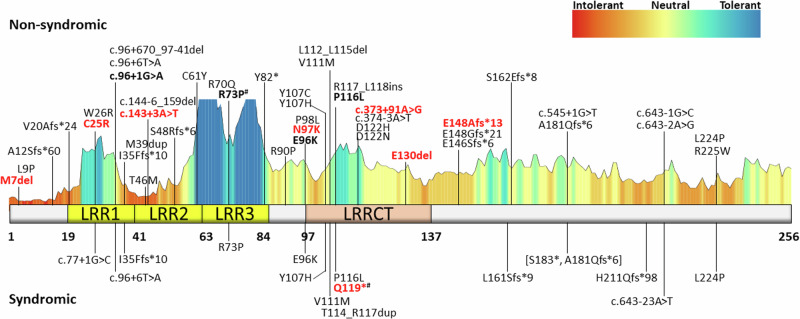
CFAP410 protein structure, mutation tolerance, and protein variants. CFAP410 secondary structure and distribution of known and novel disease variants found in affected individuals. Prediction model of the mutation tolerance landscape of the CFAP410 protein was retrieved from MetaDome webpage. Protein motifs and catalytic domain are highlighted using different colors, while variants were divided in two groups, depending whether they were found in syndromic or non-syndromic IRD patients. Known variants were retrieved from the Leiden Open Variation Database (LOVD). Variants reported in this study are in bold and novel variants are further highlighted in red. Variants p.S183* and p.A181Qfs*6, in square brackets, are part of the same complex allele as they result from the same nucleotide variant. # variants were found in mild syndromic cases. LRR, leucine-rich repeat; LRRCT, LRR C-terminal domain.

Variants in *CFAP410* have been associated with both syndromic (i.e., skeletal ciliopathies) and non-syndromic forms of retinal degeneration. To investigate whether this phenotypic variability was the consequence of a specific variant localization, we plotted the known 42 *CFAP410* variants reported in literature and the eight novel variants detected in our probands onto the secondary structure of the human CFAP410, a 256 amino acid protein (UniProtKB ID: O43822) (see Fig. 4 and Supplementary Table 3)^6,9–16,18,19,24,25,29–32,34,45^. Half of the 50 analyzed *CFAP410* variants were missense, while the other half were either truncating or non-coding variants. Most of the variants were located in the N-terminal half of the protein, up to the amino acid residue 130, containing three lucine-rich repeat domains (LRR) and a leucine-rich repeat C-terminal domain (LRRCT) (see Fig. 4). The mutation tolerance at CFAP410 protein residues was analyzed using MetaDome (https://stuart.radboudumc.nl/metadome/)^46^, while the impact of specific missense variants on CFAP410 structure and function was predicted by tools like SIFT^47^, PolyPhen2^48^, CADD Phred^49^ REVEL^50^, and EVE^51^. Our analyses did not reveal variants in specific regions of the protein that would explain the observed phenotypic difference between syndromic and non-syndromic cases (see Fig. 4). Nine of the *CFAP410* variants detected in syndromic cases were also found in non-syndromic cases, while six were exclusive (see Fig. 4). These were: (1) p.Leu161Serfs*9, detected in one family with severe skeletal abnormalities consistent with JS^6^; (2) c.643-23A>T, detected homozygously in five pedigrees with JS^12^, SMDAX^9^, or other forms of skeletal dysplasia^15^; (3) p.Gln119* found in the mildly syndromic proband of family 16; (4) c.77+1G>C found in a severe syndromic IRD case (LOVD data and personal communication); (5) p.Thr114_Arg117dup, detected homozygously in a milder^17^ syndromic IRD patient; and (6) p.His211Glnfs*98 found in one case with SMDAX and CRD^11^ (see Fig. 4 and Supplementary Table 2).

**Fig. 5:**
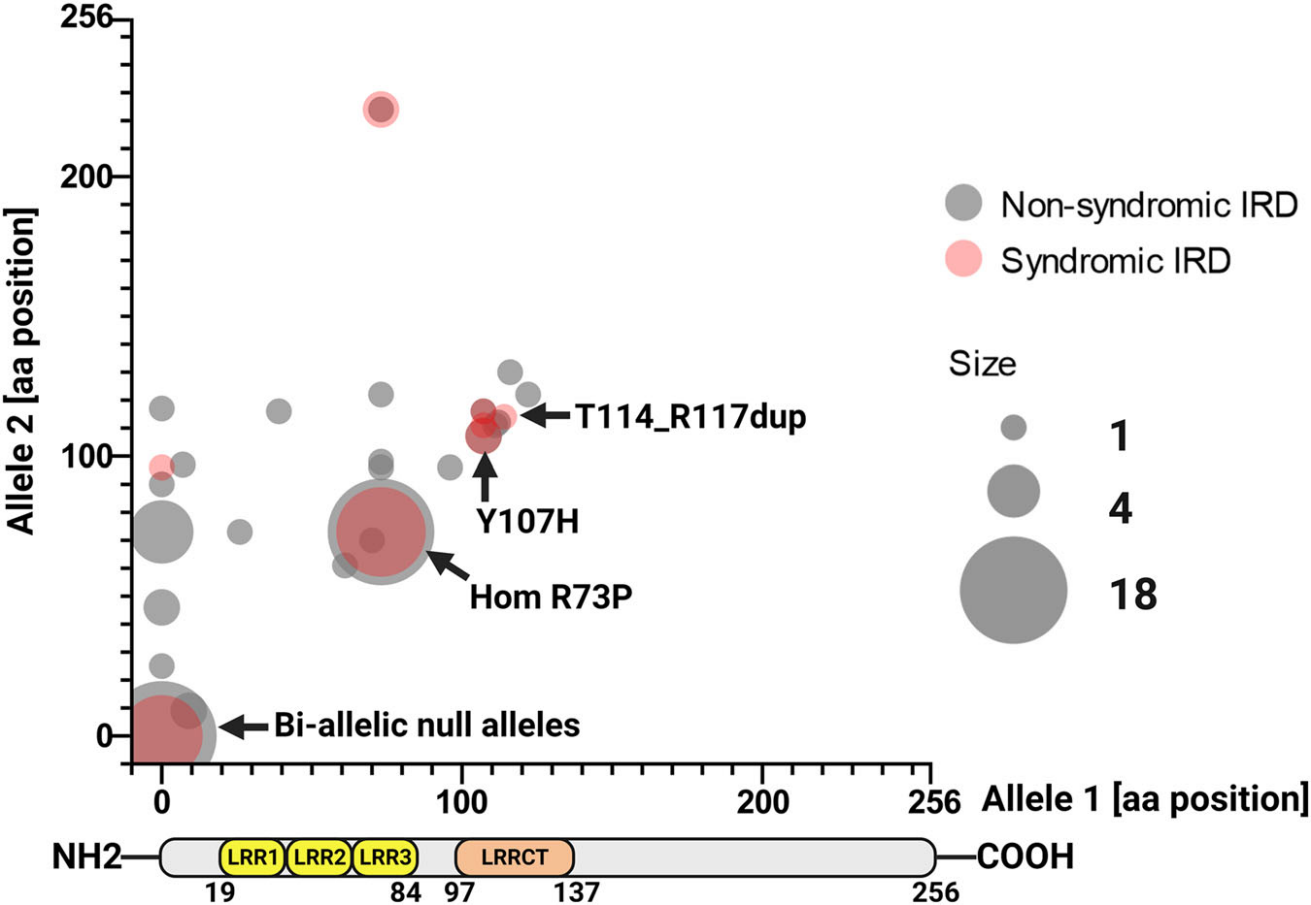
Genotype-phenotype correlation of 95 syndromic and non-syndromic patients carrying bi-allelic variants in *CFAP410.* For each patient, variants on alleles 1 and 2, represented as protein changes, were plotted on x and y axes. Predicted loss-of-function variants were represented as null (zero). Two larger clusters were found for cases homozygous for null alleles and for the p.Arg73Pro change, while a minor cluster included variants located in the LLRCT domain, in particular p.Tyr107His and p.Thr114_Arg117dup. Syndromic phenotype was presented in red and non-syndromic phenotype was presented in gray.

After plotting genotypes of 95 cases carrying bi-allelic *CFAP410* variants, including all reported and our probands (see Fig. 5), we did not observe a clear correlation between the severity of the phyenotype (syndromic vs non-syndromic) and the variants. Most of the cases in both cohorts were either bi-allelic for the loss of function variants or were homozygous for the p.Arg73Pro variant. Other missense variants, mostly affecting the LRR and LRRCT domains were also present in syndromic and non-syndromic IRD cases (see Fig. 5). There was also no apparent correlation between a specific retinal phenotype and *CFAP410* variants (see Supplementary Table 2).

Adopting the American College of Medical Genetics (ACMG) guidelines^52^, ten of the identified *CFAP410* variants were classified as pathogenic/likely pathogenic while p.Met7del and p.Asn97Lys were classified as variants of uncertain significance (VUSs) (Table 2).

## Discussion

In this retrospective study, we describe sixteen probands with retinal degeneration associated with rare bi-allelic variants in *CFAP410*, a gene initially associated with recessive skeletal ciliopathies like JS and SMDAX. Fourteen probands in our cohort did not have any syndromic features, and two individuals were recognized to have systemic findings related to *CFAP410* variants, noted only after genetic testing was performed. Other bi-alleleic *CFAP410* cases were described in the literature with non-syndromic or mildly syndromic IRDs^10–13,19–35^. Our study thus further supports the association of variants in *CFAP410* with non-syndromic IRDs first described by Khan and colleagues^18^ and considerably increases the number of non-syndromic cases.

This report also expands upon prior reports of *CFAP410*-associated retinopathy, as cases presented here exhibited a spectrum of clinical diagnoses with CRD and RCD equally represented. Both patient-reported symptoms and assessments of retinal function segregated into these different diagnostic categories and supported the differing ways in which *CFAP410* dysfunction can manifest. A notable feature, regardless of clinical diagnosis, was the early disease onset, with symptoms beginning prior to the age of 10 years in those for whom a specific age could be recalled. Two-thirds of the 36 patients described by Shinbashi et al. had symptom onset before age 18^53^. An additional aspect emphasized by the present cohort is the severity of central vision loss independent of clinical diagnosis: except probands of families 10 and 11, no other individuals in our cohort, including three between the ages of 9 and 13, had visual acuity better than 20/80 at the time of evaluation in our clinics. Indeed, the nystagmus observed in two patients, one with eoRD and one with CRD, is consistent with the early presence of central visual impairment. In the eight probands for whom spectral domain OCTs could be digitally reviewed, the degree of visual impairment was noted to be disproportionate to the degree of structural disruption. That is, although foveal structure was not normal in any of these patients, better visual acuity might have been anticipated. Posterior staphylomas disproportionate to the degree of myopia were present in three individuals, as previously reported by Khan and others^18^.

We identified eight novel variants, six of which are pathogenic/likely pathogenic, including the non-coding variant c.373+91A>G, which causes splicing defect and premature transcript truncation. Despite the spectrum of clinical variation, no genotype-phenotype correlations could be identified with regard to retinal phenotype.

The most recurrent variant in our cohort was the previously reported p.Arg73Pro change, found in eight homozygotes and two heterozygotes across clinical diagnoses. This variant is by far the most frequent change detected in *CFAP410* patients (see Supplementary Table 3) and it is the only described pathogenic variant localizing in the third leucine-reach repeat domain, although very recently a similar missense variant located closeby but classified as VUS (p.Arg70Gln) has been detected homozygously in one CRD case^26^. Its total allele frequency is 0.0005023 in GnomAD v4, largely enriched in non-Finnish Europeans. The common origin for our cases carrying the p.Arg73Pro variant were Brittany and the British isles, particularly Ireland, suggesting a possible founder allele. The p.Arg73Pro variant is associated with a broad phenotypic spectrum (see Fig. 1)^6,9,14,20,22,24–27,29,32,33,37^. The proband from family 14, who was homozygous for the p.Arg73Pro variant, had thoracic skeletal abnormalities for which two surgeries were required. Homozygosity for the p.Arg73Pro variant has also been reported previously in JS, SMDAX, and other syndromic IRD cases^6,9,14^. However, six additional probands in our cohort, homozygous for the p.Arg73Pro variant, lacked extraocular features.

Proband from family 16, homozygous for the p.Gln119* change, suffered from bilateral hip dysplasia, asymmetric bilateral hearing loss, and early ovarian failure. The p.Gln119* change introduces a stop codon in exon 4, of the 7 exon *CFAP410* gene, which most likely leads to nonsense-mediated decay (NMD)^54–58^ of the whole transcript and thus is considered a null allele. Since proband from family 16 does not have any functional CFAP410 protein, we consider her overall phenotype to be relatively mild compared to JS cases^7,8^. The other truncating variant detected in this study, p.Glu148Alafs*13, is located in exon 5 and is also thought to lead to transcript degradation through NMD and thus a null allele. This variant appeared in *trans* with the p.Arg73Pro change in the non-syndromic proband of family 4. Such genotypes were also reported in the past to lead to more severe phenotypes^6,9,12,14,15^.

The two non-coding variants validated in our study, c.143+3A>T and c.373+91A>G, showed a full and partial splicing defect on a mini-gene splicing assay, respectively. Both cases presented with a non-syndromic retinal degeneration (see Fig. 1 and Supplementary Fig. 6). It is important to mention that under the experimental settings of our splicing assay, namely testing the effect of a variant in a limited genomic context, the strength of the observed splicing effect is approximate and we cannot rule out that the c.143+3A>T might have a less severe molecular effect when tested in a larger genomic context.

A review of 95 previously published and our bi-allelic *CFAP410* cases did not reveal a clear genotype-phenotype corrlelation and even suggested that a non-syndromic retinal degeneration is likely more common than the syndromic IRD/skeletal dysplasia in patients affected by variants in this gene. Since the actual function of CFAP410 protein remains unknown, it is still unclear what are the molecular mechanisms able to explain the phenotypic heterogeneity observed in patients carrying mutations in this gene. It has been hypothesized that this variability might be the consequence of the functional interaction of CFAP410 with two other proteins NEK1 and SPATA7, as they form a protein complex localized to photoreceptor ciliary structures in multiple species including humans^6,13,18^. Therefore, it seems likely that this protein complex might have different targets, some of which tissue-specific, eventually resulting in different clinical signs^6,9,59^. We hypothesize that other proteins may be able to partially substitute for the CFAP410 protein function, which can be facilitated by modifying variants in genes encoding these proteins and thus influencing disease severity and progression^60^. Such variants have been described in other ciliopathy cohorts, for example, the *AHI1* variant p.(Arg830Trp), which increases seven-fold the relative risk of retinal degeneration within a nephronophthisis cohort^61^. Similarly, resequencing of *TTC21B* gene in a large group of clinically diverse ciliopathies showed that variants in this gene account as severity modifiers in ~5% of ciliopathy patients^62^. Collaborative resequecning of all of the published and unpublished cases may reveal such genetic modifiers of the severity of *CFAP410*-associated disease in the future.

In conclusion, our data validate the phenotypic expansion caused by pathogenic variants in *CFAP410* and expand the mutation landscape of this gene by providing novel coding and non-coding variants in this ciliopathy gene.

## Methods

### Ethics statement

The study was approved by the institutional review board of all participating institutions (Committees of Protection of Persons Ile de France V for families 6, 10, 11, and 12, and Partners HealthCare System for all remaining families) and adhered to the Declaration of Helsinki. Informed consent was obtained from all individuals on whom genetic testing and further molecular evaluations were performed.

### Clinical evaluation

Sixteen probands with autosomal recessive retinal degeneration were enrolled in this study. Twelve of them were ascertained from Massachusetts Eye and Ear, and other four from the National Reference Centre of Rare Diseases at Quinze-Vingts National Hospital.

Clinical evaluation was performed by experienced ophthalmologists according to previously published protocols and included functional and structural assessments^63–66^.

### Genetic analysis

All probands analyzed in this study, except the ones of families 6, 10, 11, 12, and 16, are part of a historical cohort that underwent clinical evaluation in the Inherited Retinal Disorder Service (at MEE; Boston, MA) in the 1990s and early 2000s. Blood samples were obtained from probands, and when possible, their parents. DNA was isolated from peripheral blood lymphocytes by standard procedures. Probands of four families (5, 9, 13, 15) were sequenced using the Genetic Eye Disease (GEDi) panel, described previously^67,68^. The GEDi version used in this study (v6) targeted exons of 327 known and candidate IRD genes (see Supplementary Table 4)^69^. The NGS data from the GEDi panel was analyzed using Genome Analysis Toolkit (GATK) version 3^70^ and annotated using the Variant Effect Predictor tool^71^ with additional annotations taken from the gnomAD^38^, the Genomic Evolutionary Rate Profiling (GERP)^72^, SIFT^47^, PolyPhen2^48^, CADD Phred^49^ and retinal expression^73^. To detect possible copy number variations, gCNV software was used as before^74^. Relatedness of the families sequenced with GEDi panel was excluded using Peddy^75^. Exome sequencing (ES) for six probands was performed at the Center for Mendelian Genomics at the Broad Institute of the Massachusetts Institute of Technology and Harvard using methodology described previously^68^. WES data were aligned to hg38, and variants were called using the GATK HaplotypeCaller package version 3.5 (https://software.broadinstitute.org/gatk/). Data were displayed and analyzed with an online tool (https://seqr.broadinstitute.org)^76^. Genome sequencing for proband of family 3 was performed at the genomics core of the Ocular Genomics Institute. One microgram of genomic DNA purified from whole blood was fragmented to 350 bp using Covaris LE220-plus focused-ultrasonicator, followed by library preparation with KAPA HyperPrep PCRfree Kit (Roche Sequencing Solutions). Libraries were multiplexed by adding 10 bp indexes during adapter ligation (IDT for Illumina—TruSeq DNA UD Indexes v2). Library quality was assessed by fluorometric and fragmentation analysis prior to sequencing. Paired-end 150 cycle sequencing for a minimum of 30x depth of coverage was performed on a NovaSeq 6000 (Illumina).

Probands from families 6, 10, 11, and 12 had been screened applying a customized NGS panel as reported before^77^ updated regularly to include newly IRD-associated genes, while NGS-based testing was performed by commercial diagnostic laboratories for the proband in Family 16.

### Variant validation and phasing

All presented variants refer to the *CFAP410* transcript NM_004928.3. Variant segregation was performed by Sanger sequencing (see Supplementary Table 5) or analysis of NGS reads. Although the variants detected in probands of families 4 and 7 were considered in *trans* according to the gnomAD browser Variant Co-Occurrence tool (https://gnomad.broadinstitute.org/variant-cooccurrence), they were further phased by cloning and Sanger sequencing. Briefly, genomic DNA from the proband was amplified using Phusion (New England Biolabs) and primers spanning the region containing all variants. The amplified fragment was then cloned into the pCR2.1 plasmid, TA cloning kit (Invitrogen) and Sanger sequenced. Sanger sequencing was performed on ABI 3730xl (Applied Biosystems) using BigDye Terminator v3.1 kits (Life Technologies). Sequence analysis was done using SeqManPro (Lasergene 11, DNAStar Madison, WI, USA), in which variants were considered to be in trans when they were not present on the same clone.

### Protein modeling, prediction of missense variants, and variant classification

The mutation tolerance at CFAP410 protein residues was analyzed using MetaDome (https://stuart.radboudumc.nl/metadome/)^46^, while the impact of specific missense variants on CFAP410 structure and function, was predicted by using five prediction algorithms: SIFT^47^, PolyPhen2^48^, CADD Phred^49^ REVEL^50^, and EVE^51^. Variants were finally classified according to the (ACMG) guidelines^52^.

## Supplementary information

Supplementary Information

## Data Availability

Variants are available through dbGAP (phs001272.v1.p1 and phs002459.v1.p1) and ClinVar (accession numbers SCV004232444-SCV004232454).

## Code availability

Software used in this study: Alamut Visual Plus, version 1.7.1; Biorender; GraphPad Prism, version 10; SeqManPro (Lasergene v11); Seqr (https://seqr.broadinstitute.org/).
