# Supplementary material for: Coding and non-coding variants in the ciliopathy gene CFAP410 cause early-onset non-syndromic retinal degeneration
Source: NPJ Genom Med. 2024 Nov 8;9:58. doi: 10.1038/s41525-024-00439-3 (PMC11549414; doi:10.1038/s41525-024-00439-3)
Supplement: Supplementary file 1 — Supplementary Information [file 41525_2024_439_MOESM1_ESM.pdf]

Supplementary Figure 1

Family 4 - CRD

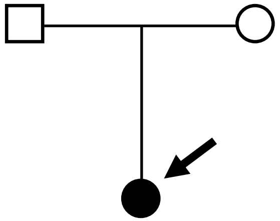

V1: c.218G>C; p.(Arg73Pro)
V4: c.441_444del; p.Glu148Alafs*13

Genotype Counts

		21-45751826-CCTC...-C		
		CCTC.../CCTC...	CCTC.../C	C/C
21-45753071-C-G	C/C	122,508	1	0
	C/G	75	0	0
	G/G	0	0	0

- Samples consistent with variants appearing in isolation or on different haplotypes.
- Samples consistent with variants appearing on the same haplotype.
- Samples consistent with either co-occurrence pattern.

Based on their co-occurrence pattern in gnomAD, these variants are likely found on different haplotypes in most individuals in gnomAD.

Note Because no individual in gnomAD carries both variants, this table was computed based on the separate variant information and does not account for the possibility that some samples may not be covered at both variant sites.

Variant 1 (required)
21-45753071-C-G

Variant 2 (required)
21-45751826-CCTCT-C

Submit

Overview

Population	Samples consistent with variants appearing in isolation or on different haplotypes	Samples consistent with variants appearing on the same haplotype	Samples consistent with either co-occurrence pattern	Likely co-occurrence pattern
African/African American	0	0	0	No prediction*
Latino/Admixed American	2	0	0	Different haplotypes
Ashkenazi Jewish	0	0	0	No prediction*
East Asian	0	0	0	No prediction*
European (Finnish)	2	0	0	No prediction*
European (non-Finnish)	69	0	0	No prediction*
Other	3	0	0	No prediction*
South Asian	0	0	0	No prediction*
**All**	**76**	**0**	**0**	**Different haplotypes**

* A likely co-occurrence pattern cannot be calculated in some cases, such as when only one of the variants is observed in a population.

Haplotype Counts

		21-45751826-CCTC...-C	
		CCTC...	C
21-45753071-C-G	C	245,092	1
	G	75	0

The estimated probability that these variants occur in different haplotypes is 100%.

Note Probability values are not well calibrated, particularly where both variants are extremely rare. Interpret with caution. Please see our blog post on variant co-occurrence for accuracy estimates and additional detail.

Supplementary Figure 1: . Confirmation of the *trans* configuration of the *CFAP410* variants V1 and V4 in proband of family 4, according to the gnomAD v2 Variant Co-Occurrence tool.

Supplementary Figure 2

Family 7 - CRD

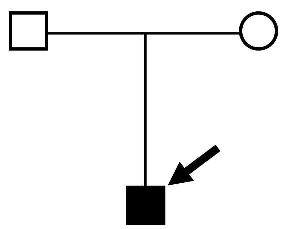

[V8];[V9]

V8: c.347C>T; p.(Pro116Leu)
V9: c.388_390del; p.(Glu130del)

Genotype Counts

		21-45751880-GCTC-G		
		**GCTC/GCTC**	**GCTC/G**	**G/G**
	**G/G**	116,843	1	0
**21-45752942-G-A**	**G/A**	14	0	0
	**A/A**	0	0	0

- Samples consistent with variants appearing in isolation or on different haplotypes.
- Samples consistent with variants appearing on the same haplotype.
- Samples consistent with either co-occurrence pattern.

Based on their co-occurrence pattern in gnomAD, these variants are likely found on different haplotypes in most individuals in gnomAD.

Note Because no individual in gnomAD carries both variants, this table was computed based on the separate variant information and does not account for the possibility that some samples may not be covered at both variant sites.

Variant 1 (required)
21-45752942-G-A

Variant 2 (required)
21-45751880-GCTC-G

Submit

Overview

Population	Samples consistent with variants appearing in isolation or on different haplotypes	Samples consistent with variants appearing on the same haplotype	Samples consistent with either co-occurrence pattern	Likely co-occurrence pattern
African/African American	1	0	0	No prediction*
Latino/Admixed American	7	0	0	No prediction*
Ashkenazi Jewish	0	0	0	No prediction*
East Asian	4	0	0	No prediction*
European (Finnish)	1	0	0	No prediction*
European (non-Finnish)	0	0	0	No prediction*
Other	0	0	0	No prediction*
South Asian	2	0	0	Different haplotypes
**All**	**15**	**0**	**0**	**Different haplotypes**

* A likely co-occurrence pattern cannot be calculated in some cases, such as when only one of the variants is observed in a population.

Haplotype Counts

		21-45751880-GCTC-G	
		**GCTC**	**G**
**21-45752942-G-A**	**G**	233,701	1
	**A**	14	0

The estimated probability that these variants occur in different haplotypes is 100%.

Note Probability values are not well calibrated, particularly where both variants are extremely rare. Interpret with caution. Please see our blog post on variant co-occurrence for accuracy estimates and additional detail.

Supplementary Figure 2: . Confirmation of the *trans* configuration of the *CFAP410* variants V8 and V9 in proband of family 7, according to the gnomAD v2 Variant Co-Occurrence tool.

Supplementary Figure 3

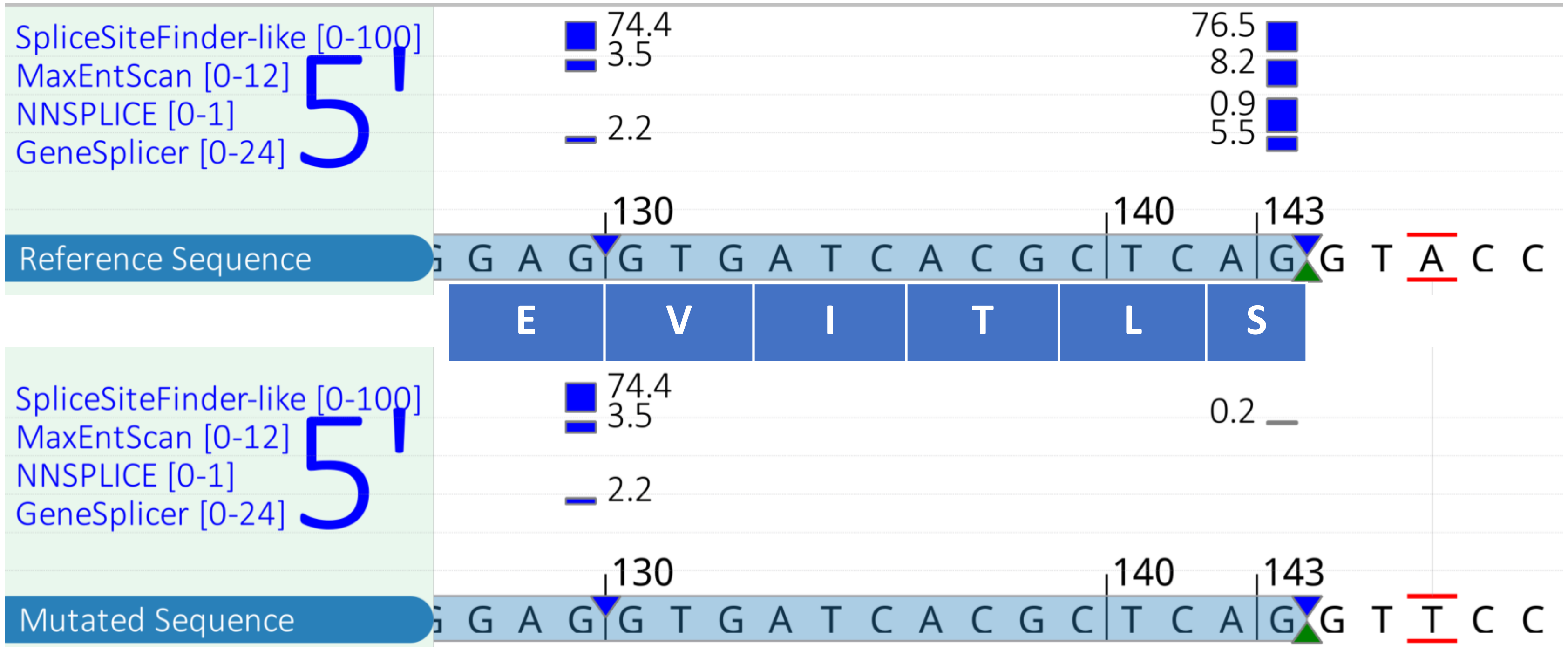

Supplementary Figure 3: Splicing prediction for *CFAP410* c.143+3A>T on intron 3. Exon 3 has an alternative splice donor site, which could be used when the canonical site is disrupted by the mutation. However, both partial or full exon 3 skipping will lead to a premature stop codon and loss-of function, as this 47-bp exon is not in frame, and the position of the alternative splice donor site does not re-establish the frame as well.

Supplementary Figure 4

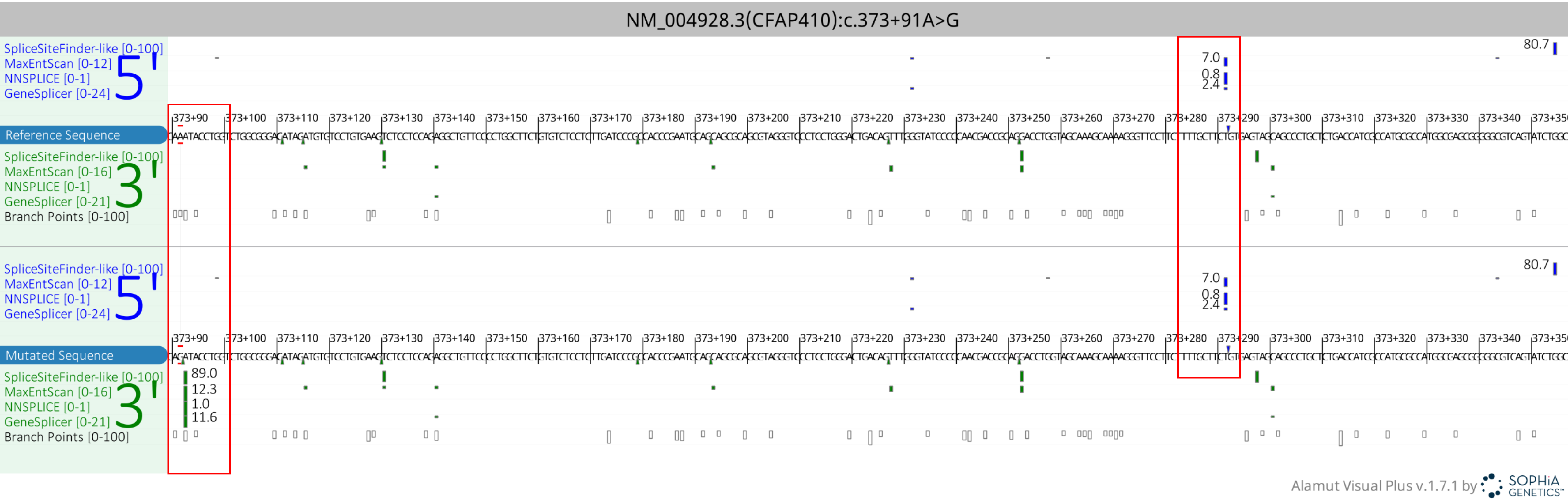

Supplementary Figure 4: Splicing prediction for *CFAP410* c.373+91A>G on intron 4. The creation of a new cryptic slice acceptor site and the presence of a downstream splice donor site lead to a cryptic exon recognition and consequent missplicing event.

Supplementary Figure 5

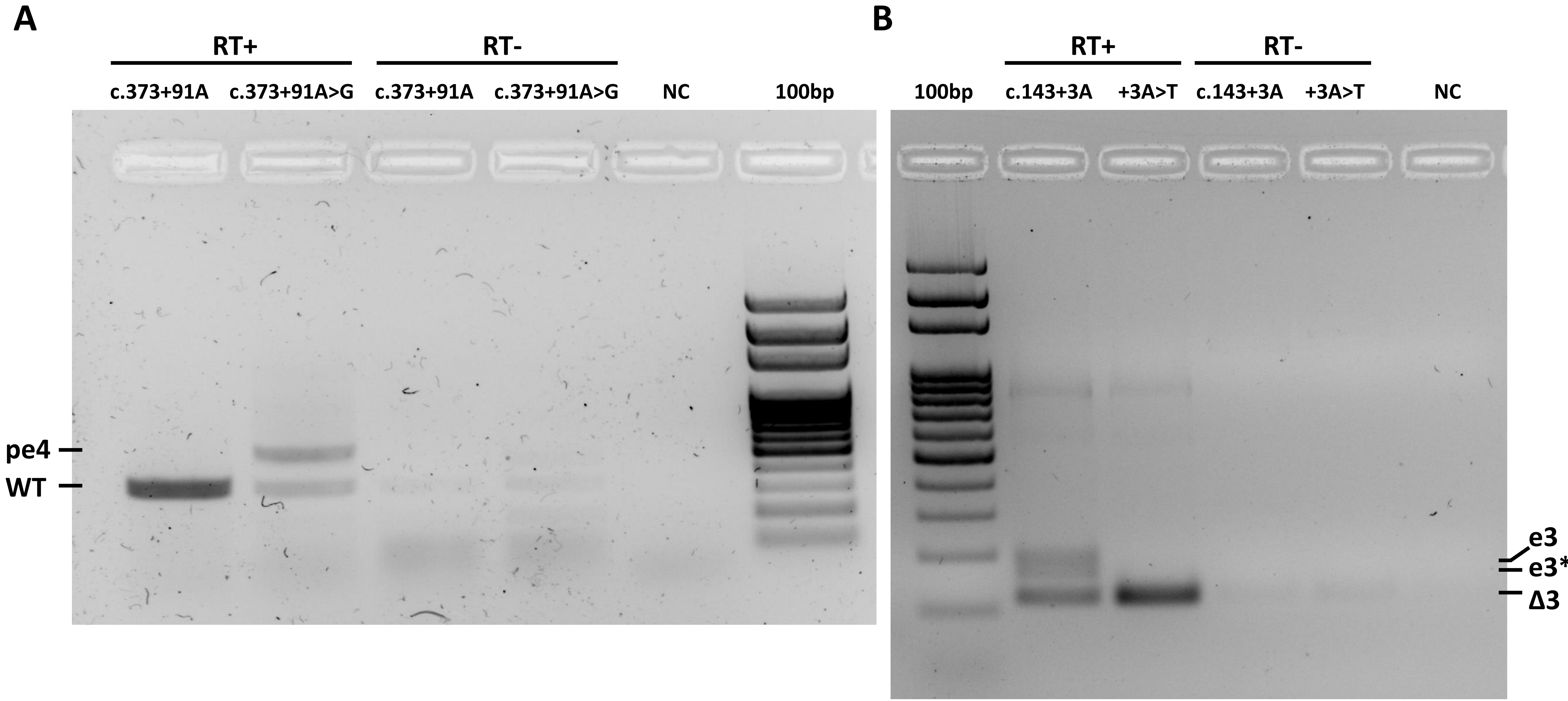

Supplementary Figure 5: Uncropped and unprocessed scans of gel blots showed in Figure 3

Supplementary Figure 6

Family 3 - CRD

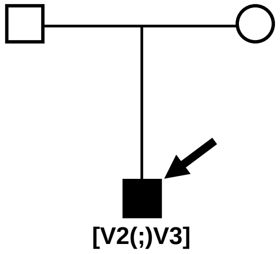

V2: c.73T>C; p.(Cys25Arg)
V3: c.373+91A>G

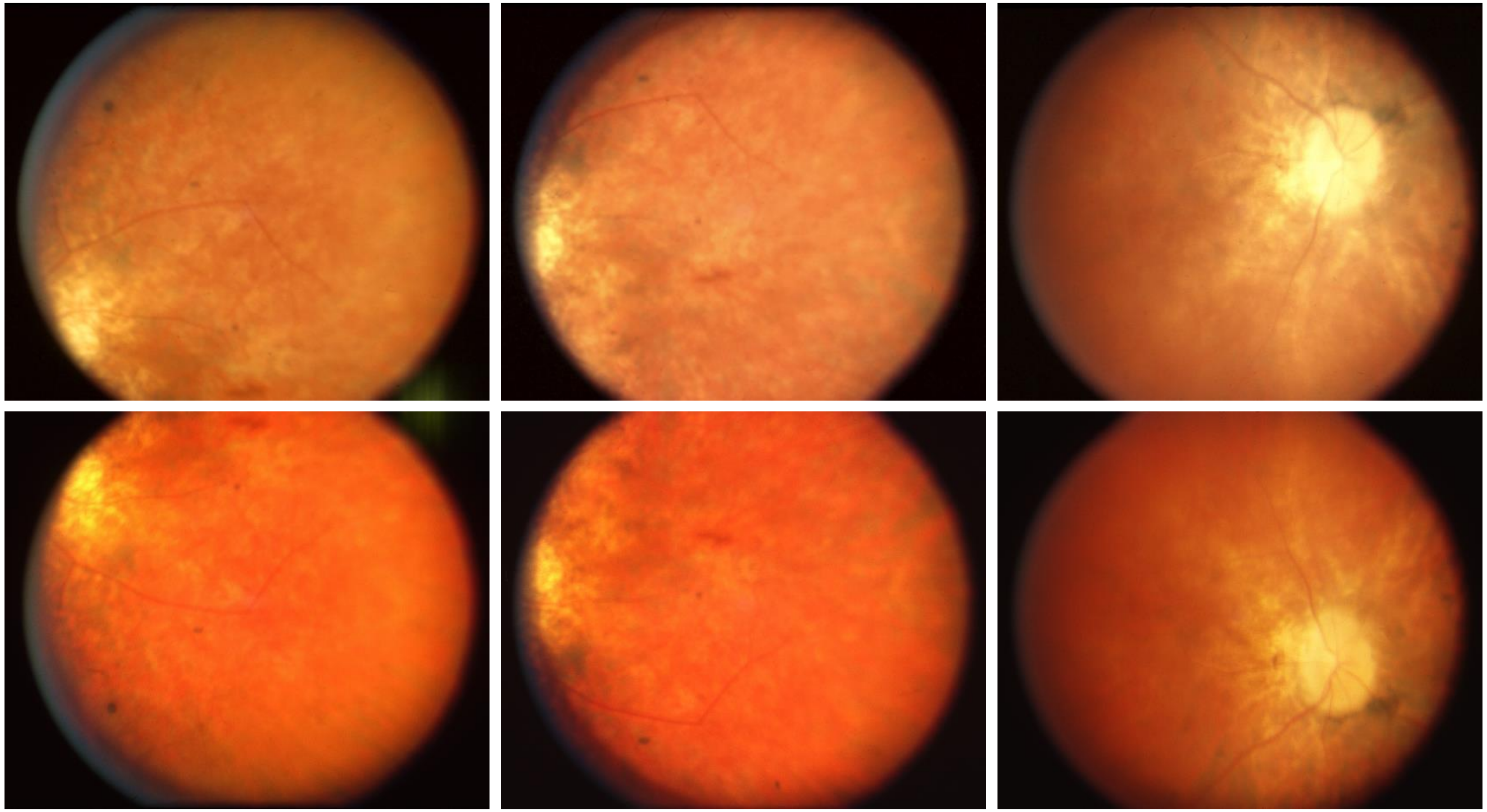

Supplementary Figure 6: Fundus pictures of proband of family 3.

Family_ Proband	Proband_ Research_ID	Age at initial visit; Gender	Dx	Ethnicity	Non-retinal features	Ocular symptoms	VA	Visual field*	ERG	Fundoscopy	FAF	OCT	Refractive error	Other ocular findings/history
1_II-1	OGI3083_4678	13 | M	CD	White (Scottish/ Irish/ Italian)	None$	Light sensitivity, ↓ central vision	20/100 OD 20/200 OS	OU: Full to I4e and V4e	Scotopic dim flash: NL Scotopic bright flash: 75% NL OD, NL OS 30 Hz flicker: <2% NL; delayed	Disc, vessels, and periphery within normal limits, macular granularity	n.a.	Reduced foveal thickness; limited interpretation possible	OD +1.25 -2.50 x005 OS +0.75 -2.25 x175	n.a.
2_II-1	OGI3014_4600	25 | F	CRD	White# (Irish)	None$	Poor vision since age 6; photosensitivity	10/200 OD 15/200 OS<br>Age 37: 20/200 OD 20/400 OS	OU: Relative central scotoma to I4e; otherwise full	Scotopic dim flash: 70-75% NL Scotopic bright flash: 70-91% NL 30 Hz flicker: ~6% NL, delayed Age 37: Scotopic dim flash: 30-40% NL Scotopic bright flash: 50-65% NL 30 Hz flicker: <5% NL; delayed	Normal nerve color; attenuated vessels; granular-appearing macular and peripheral pigmentation	n.a.	n.a.	OD -2.0 sph OS -1.75 +1.75 x180	Nystagmus noted on exam.
3_II-1	OGI1446_2641	32 | M	CRD	White (Italian)	Premature birth, no skeletal abnormalities reported or seen on extensive x-rays.	↓ central vision at age 6, ↓ color vision at age 18, ↓ night vision at age 19	CF OU<br>Per outside records: Age 6: 20/60 OU Age 18: 20/80 OU	n.a.	Scotopic dim flash: 5% NL Scotopic bright flash: 20% NL 30 Hz flicker: n.d.	Pale elliptical nerves with temporal crescent OS. Attenuated vessels, prominent choroidal pattern with scattered peripheral spiculation	n.a.	n.a.	OD -5.0 -2.50 x020 OS -5.0 -2.50 x170	n.a.

4_II-1	OGI3006_4592	22 | F	CRD	n.a.	None[$]	↓ central vision since childhood; ↓ peripheral vision in teens	20/80 OU	OU: I4e pericentral scotomas sparing central 5-10°; V4e full	Scotopic dim flash: 30-40% NL Scotopic bright flash: <40% NL 30 Hz flicker: <5% NL; delayed	Normal nerve; attenuated vessels; preservation of central macular pigment with surrounding atrophy; peripheral granularity without bone spicules	n.a.	Normal central macular thickness; available images do not permit additional comment on outer retinal structure	OD +2.5 -2.25 x065 OS +4.0 -2.25 x180	Staphyloma
5_II-1	OGI3900_53031	9 | M	CRD	Japanese	None[$]	↓ vision at age 1	20/100 OD 20/125 OS	n.a.	Outside study at age 7: Severely reduced photopic>scotopic responses	Normal nerve; subtle vessel attenuation; macula unremarkable; midperipheral tapetal-like sheen on photos; no bone spicules	OU: Foveal hyperAF; perifoveal hyperAF ring; areas of abnormal peripheral hypo- and hyperAF	OU: Blurred defintion of outer retinal bands centrally with attenuation in peripheral macula	At age 7: OD +5.0 -3.0 x180 OS +5.5 -3.25 x180	n.a.
6_II-1	CIC07923_F4428	51 | M	CRD	Black (Senegalese)	None[$]	Diagnosis in childhood of nyctalopia and severely decreased VA	OU 20/160	OU III4e ~5°	decreased scotopic responses 50% of NL, n.d. photopic responses	Ovoid pale optic disc, narrowed retinal vessels, peripheral granular chorioretinal atrophy with sparse pigment migration, bull's eye maculopathy	Patchy loss of AF outside the vascular arcades; perifoveal hyoautofluorescence with a bull's eye appearance	Myopic staphyloma, preserved ONL at the fovea but absence of hyepr reflective bands, loss of outer retinal layer in the periphery	OD -9,50(-1,25)90° OS -11,50'-0,75)85°	none
7_II-1	OGI3057_4647	45 | M	CRD	Indian	None[$]	↓ central vision since age 18 and night vision since age 30, delayed dark adaptation	CF OD 20/200 OS	OD: sees V4e only OS: mild generalized constriction to I4e & V4e	Scotopic dim flash: n.d. Scotopic bright flash: <20% NL 30 Hz flicker: <2% NL, delayed	n.a.	n.a.	n.a.	n.a.	n.a.

8_II-1	OGI2139_ 3573	38 | F	RCD	White (English/ Irish)	None[$]	↓ central vision since age 7; ↓ night and peripheral vision since age 28	20/80 OU<br><br>Age 56: 20/250 OD 20/300 OS	Constriction of I4e to ~12° OU and V4e to 90-95° OU	Scotopic dim flash: n.d. Scotopic bright flash: <10% NL 30 Hz flicker: <2% NL; delayed	Myopic tilted nerve, peripheral atrophy, vessel attenuation, macular and peripheral granularity with sparse bone spicules<br>Age 56: Tilted nerve, staphyloma, central macular atrophy, peripheral atrophy (anterior>>midperipheral) with small round pigment clumps	Age 56: Central macular hypoAF; granular peripheral hypoAF	Age 56:Outer retinal bands present but poorly defined at fovea and nasally; absent elsewhere	OD -3.75 -1.75 x160<br>OS -2.25 -2.00 x015	Age 56: Posterior staphyloma OU
9_II-1	OGI1877_3254	12 | F	RCD	White (Irish)	None[$]	Nyctalopia and ↓ peripheral vision since age 4	20/100 OU	OD: I4e ~ 5°, V4e ~20°<br>OS: no I4e sensitivity, V4e ~20°	Scotopic dim flash: n.d. Scotopic bright flash: n.d. 30 Hz: <2% NL, delayed	Pale nerves, attenuated vessels, macular and peripheral granularity	n.a.	n.a.	OD -4.00 -2.50 x016<br>OS -4.00 -2.50 x153	n.a.
10_II-1	CIC04687_F2325	38 | M	RCD	White (Breton)	None[$]	Nyctalopia since early childhood, never had VA above 20/32, progressive visual field constriction, diagnosis of RCD at 14	20/40 OU	OU II4e ~ 5°, V4e ~10°	n.d for both scotopic and photopic conditions	Ovoid pale optic disc, peripapillary atrophy, narrowed retinal vessels, peripheral granular chorioretinal atrophy with sparse pigment migration, perifoveal atrophy	Patchy loss of AF in the periphery; perifoveal ring of loss of AF	Myopic staphyloma, preserved ONL at the fovea but hyporeflectivty of EZ and IZ, loss of outer retinal layer in the periphery	OD-3.25(-0.50)140°<br>OS -3.25(-0.75)170°	none
11_II-1	CIC03728_F1669	63 | F	RCD	White (Breton)	None[$]	Nyctalopia since teens, progressive visual field constriction, diagnosis of RCD in her 20s	OD 20/50 OS 20/40	OU IV4e ~90°	n.d for both scotopic and photopic conditions	Ovoid pale optic disc, narrowed retinal vessels, peripheral granular chorioretinal atrophy with sparse pigment migration, perifoveal atrophy	Patchy loss of AF outside the vascular arcades; perifoveal ring of loss of AF	n.a.	OD +2,25(-0;75)45°<br>OS +1	bilateral pseudophakia, was myopic before

12_II-1	CIC01570_ F4189	71 | F	RCD	White (Breton)	None[$]	Diagnosis in childhood of nyctalopia and progressive field constriction	OD 20/800<br>OS 20/400	OU III4e ~5°	n.d for both scotopic and photopic conditions	Optic nerve drusen, narrowed retinal vessels, peripheral granular chorioretinal atrophy with sparse pigment migration	Optic nerve drusen, patchy loss of AF outside the vascular arcades	Myopic staphyloma, preserved ONL at the fovea but hyporeflectivty of EZ and IZ, loss of outer retinal layer in the periphery	OD +2,50(-2)15°<br>OS +4(-1)100°	bilateral pseudophakia, was myopic before
13_II-1	OGI1309_ 2454	57 | M	RCD	White	None[$]	Nyctalopia and ↓ peripheral vision since childhood	LP OD<br>20/200 OS<br><br>Age 61: LP OU	OD: n.a.<br>OS: intermittent central sensitivity to V4e only	Scotopic dim flash: 10% NL<br>Scotopic bright flash: <2% NL<br>30 Hz: <2% NL; delayed	Pale nerve, attenuated vessels, preserved central macular pigmentation, peripheral nummular chorioretinal atrophy with scant pigmentary deposits	OD: Relative preservation of macular AF; peripheral hypoAF<br><br>OS: Parafoveal hyperAF; peripheral hypoAF	OD: bare ELM at fovea with surrounding outer retinal atrophy<br>OS: ELM and bare EZ subfoveally with surrounding outer retinal atrophy	n.a.	Amblyopia OD; Posterior staphyloma OU on OCT
14_II-1	OGI1369_ 2543	32 | M	RCD	White (Scottish)	Thoracic skeletal abnormalities[$$]	Nyctalopia since childhood	20/200 OD<br>20/600 OS	OU: V4e only; ~15° centrally plus isolated peripheral islands	Scotopic dim flash: n.d.<br>Scotopic bright flash: n.d. OD, <2% NL OS<br>30 Hz: <2% NL; delayed	Myopic-appearing fundus. Pale nerve; attenuated vessels; midperipheral atrophy with sparse bone spicules and pigment deposits	OU: Abnormal macular hypoAF. Ring of hypoAF centered on arcades; also anterior peripheral hypoAF	OU: Diffuse outer retinal atrophy with outer retinal bands (ONL, ELM) visible only at fovea	SE -6.5 OD, -7.0 OS	History of strabismus surgery
15_II-1	OGI2251_ 3735	46 | M	RCD	Black (Haitian[##])	None[$]	Nyctalopia since age 4, ↓ peripheral vision since age 25 and central vision since age 36	20/200 OD<br>20/400 OS	OU: V4e only; ~10° centrally	Scotopic dim flash: n.d.<br>Scotopic bright flash: n.d.<br>30 Hz: <2% NL, delayed	Waxy pallor nerve, macular granularity, attenuated vessels, bone spicule pigmentation peripherally	n.a.	n.a.	OD -3.50 -2.00 x040<br>OS -4.00 -3.00 x105	n.a.

16_II-1	OGI3844_ 52295	50 | F	eo-RD	White	Bilateral hip dysplasia, asymmetric bilateral hearing loss (40s), early ovarian failure (30)	Nystagmus as infant. Nyctalopia since early childhood. Never 20/20; ↓ central vision in 20s. ↓ peripheral vision in 40s	20/150 OD<br>LP OS	OD only: Island of inferotemporal midperipheral I4e sensitivity; V4e near-full	Scotopic dim flash: n.d.<br>Scotopic bright flash: <5% NL OD, n.d. OS<br>30 Hz: <2-5% NL, delayed	OD: Macula unremarkable. Vascular attenuation. Inferior atrophy with sparse pigment clumps and bone spicules.<br><br>OS: Morning glory disc. Attenuated vessels. Macular atrophy. Peripheral atrophy, bone spicules, and pigment clumps with relative superior and mid-temporal sparing.	OD: Subtle parafoveal hyperAF; granular hypoAF peripherally inferior>>elsewhere<br><br>OS: HypoAF throughout macula and posterior pole extending inferiorly; areas of superior and nasal sparing	OD: Poorly defined EZ band visible only at fovea and parafoveally; ONL thin throughout.<br>OS: Diffuse absence of outer retinal laminae.	OD pseudophakic<br>OS -1.50 -1.50 x093	Morning glory disc OS; Nystagmus

Supplementary_Table_1: Clinical characteristics of IRD probands carrying *CFAP410* variants. AF, autofluorescence; CF, counting fingers; CD, cone dystrophy; CRD, cone-rod dystrophy; Dx, diagnosis (supported by ERG findings); eoRD, early-onset retinal dystrophy; ELM, external limiting membrane; ERG, electroretinogram; EZ, ellipsoid zone; F, female; FAF, Fundus autofluorescence; Hz, Hertz; IZ, Interdigitation zone; M, male; LP, light perception; n.a., not available; n.d., not detected; NL, normal; OCT, optical coherence tomography; ONL, outer nuclear layer; OD, right eye; OS, left eye; OU, both eyes; RCD, rod-cone dystrophy; VA, visual acuity; #, parents are third cousins; ##, parents are first cousins; $, no skeletal abnormalities on questionnaire; $$, prior rib cage procedure.

LOVD_ID	Study_ID	*CFAP410* _ allele 1 (c.)	*CFAP410* _ allele 1 (p.)	ACMG allele 1	*CFAP410* _ allele 2 (c.)	*CFAP410* _ allele 2 (p.)	ACMG allele 2	Reference	Non-syndromic diagnosis	Syndromic diagnosis
n.a.	Patient 1	c.26T>C	p.(Leu9Pro)	VUS	c.26T>C	p.(Leu9Pro)	VUS	Shinbashi 2023	RCD	
n.a.	Patient 2 (sib)	c.26T>C	p.(Leu9Pro)	VUS	c.26T>C	p.(Leu9Pro)	VUS	Shinbashi 2023	RCD	
00328082	G004999	c.33_34insAGCTGCACAGCGTGCA	p.(Ala12Serfs*60)	P	c.218G>C	p.(Arg73Pro)	LP	Carss 2017; Turro 2020	RCD	
00328142	G005543	c.33_34insAGCTGCACAGCGTGCA	p.(Ala12Serfs*60)	P	c.269G>C	p.(Arg90Pro)	P	Carss 2017; Turro 2020	CRD	
00381891	41	c.33_34insAGCTGCACAGCGTGCA	p.(Ala12Serfs*60)	P	c.374-3A>T	p.(?)	VUS	Birtel 2018	RD	
00377177	242	c.33_34insAGCTGCACAGCGTGCA	p.(Ala12Serfs*60)	P	c.33_34insAGCTGCACAGCGTGCA	p.(Ala12Serfs*60)	P	Tracewska 2021	RD	
00303489	n.a.	c.59_62del	p.(Val20Alafs*24)	P	c.443_444del	p.(Glu148Glyfs*21)	P	n.a.	RCD	
00447297	SRP-1198	c.59_62del	p.(Val20Alafs*24)	P	c.443_444del	p.(Glu148Glyfs*21)	P	Weisschuh 2024	RCD	
n.a.	n.a.	c.77+1G>C	p.(?)	P	c.77+1G>C	p.(?)	P	n.a.		Skeletal dysplasia (short stature, narrow chest) + RCD
00362086	QT803	c.96+1G>A	p.(?)	P	c.352_353insACCCTGCCGCGC	p.(Arg117_Leu118insHisProAlaAla)	LP	Huang 2016	CRD	
00386272	RPN-147	c.96+1G>A	p.(?)	P	c.96+1G>A	p.(?)	P	Rodriguez-Munoz 2020	CRD	
00386273	RPN-148 (sib)	c.96+1G>A	p.(?)	P	c.96+1G>A	p.(?)	P	Rodriguez-Munoz 2020	CRD	
00248834	FamUCL78P1	c.96+6T>A	p.(?)	LP	c.480_481insT	p.(Leu161Serfs*9)	P	Wheway 2015		JS + CRD
00327515	FamPatII4	c.96+670_97-41del	p.(?)	LP	c.96+670_97-41del	p.(?)	LP	Gustafson 2017	RCD	
00059794	n.a.	c.103del	p.(Ile35Phefs*10)	P	c.103del	p.(Ile35Phefs*10)	P	Abu-Safieh-2013	CRD	
00248836	FamCR-F024P1	c.103del	p.(Ile35Phefs*10)	P	c.103del	p.(Ile35Phefs*10)	P	Wheway 2015	CRD	
00331335	11DG0973	c.103del	p.(Ile35Phefs*10)	P	c.103del	p.(Ile35Phefs*10)	P	Maddirevula 2018		Skeletal Displasia + RD
00380303	n.a.	c.103del	p.(Ile35Phefs*10)	P	c.103del	p.(Ile35Phefs*10)	P	Abu-Safieh-2013	RCD	
00379814	2017010402	c.137C>T	p.(Thr46Met)	LP	c.144-6_159del	p.(?)	P	Wang 2018	RCD	
00393626	n.a.	c.137C>T	p.(Thr46Met)	LP	c.144_159del	p.(Ser48Argfs*6)	P	Liu-2020	RCD	
00327506	Pat3	c.182G>A	p.(Cys61Tyr)	P	c.182G>A	p.(Cys61Tyr)	P	Khan 2015	RD	
00450778	074614	c.209G>A	p.(Arg70Gln)	VUS	c.209G>A	p.(Arg70Gln)	VUS	Hitti-Malin 2024	CRD	

00248833	FamUCL-111P1 (proband)	c.218G>C	p.(Arg73Pro)	LP	c.671T>C	p.(Leu224Pro)	P	Wheway 2015		JS + CRD
n.a.	FamUCL-111P2 (sib)	c.218G>C	p.(Arg73Pro)	LP	c.671T>C	p.(Leu224Pro)	P	Wheway 2015		JS + CRD
n.a.	FamUCL-111P6 (sib)	c.218G>C	p.(Arg73Pro)	LP	c.671T>C	p.(Leu224Pro)	P	Wheway 2015	CRD	
00358801	BLM033	c.218G>C	p.(Arg73Pro)	LP	c.364G>C	p.(Asp122His)	VUS	Zhang 2016	RCD	
00389734	1018	c.218G>C	p.(Arg73Pro)	LP	c.96+6T>A	p.(?)	LP	Weisschuh 2020	RCD	
00386224	RPN-402	c.218G>C	p.(Arg73Pro)	LP	c.246C>A	p.(Tyr82*)	P	Rodriguez-Munoz 2020	CRD	
00386225	RPN-403 (sib)	c.218G>C	p.(Arg73Pro)	LP	c.246C>A	p.(Tyr82*)	P	Rodriguez-Munoz 2020	CRD	
00362223	Pat5	c.218G>C	p.(Arg73Pro)	LP	c.76T>C	p.(Trp26Arg)	P	Fadaie 2021	RCD	
00447538	CRD-803	c.218G>C	p.(Arg73Pro)	LP	c.293C>T	p.(Pro98Leu)	VUS	Weisschuh 2024	CRD	
n.a.	n.a.	c.218G>C	p.(Arg73Pro)	LP	c.482dup	p.(Ser162Glufs*8)	P	LOVD data, personal communication	CD	
00248835	FamGC4693 Pat1	c.218G>C	p.(Arg73Pro)	LP	c.218G>C	p.(Arg73Pro)	LP	Wheway 2015		JS + CRD
n.a.	FamGC4693 Pat2 (sib)	c.218G>C	p.(Arg73Pro)	LP	c.218G>C	p.(Arg73Pro)	LP	Wheway 2015		JS + CRD
n.a.	FamGC4693 Pat3 (sib)	c.218G>C	p.(Arg73Pro)	LP	c.218G>C	p.(Arg73Pro)	LP	Wheway 2015		JS + CRD
n.a.	FamGC4693 Pat4 (sib)	c.218G>C	p.(Arg73Pro)	LP	c.218G>C	p.(Arg73Pro)	LP	Wheway 2015		JS + CRD
n.a.	FamGC4693 Pat5 (sib)	c.218G>C	p.(Arg73Pro)	LP	c.218G>C	p.(Arg73Pro)	LP	Wheway 2015		JS + CRD
00327511	Fam8	c.218G>C	p.(Arg73Pro)	LP	c.218G>C	p.(Arg73Pro)	LP	Wang 2016		SMDAX + RCD
00327512	Fam9	c.218G>C	p.(Arg73Pro)	LP	c.218G>C	p.(Arg73Pro)	LP	Wang 2016		SMDAX + RCD
00327462	SKDP-144.3	c.218G>C	p.(Arg73Pro)	LP	c.218G>C	p.(Arg73Pro)	LP	McInerney-Leo 2017		JS + RD
00209023	28771251-Pat40	c.218G>C	p.(Arg73Pro)	LP	c.218G>C	p.(Arg73Pro)	LP	Lionel 2018	RD	
00328152	G006005	c.218G>C	p.(Arg73Pro)	LP	c.218G>C	p.(Arg73Pro)	LP	Carss 2017; Turro 2020	CD	
00308604	n.a.	c.218G>C	p.(Arg73Pro)	LP	c.218G>C	p.(Arg73Pro)	LP	Holtan 2020	RD	
00362222	Pat6	c.218G>C	p.(Arg73Pro)	LP	c.218G>C	p.(Arg73Pro)	LP	Fadaie 2021	RCD	
00377193	269	c.218G>C	p.(Arg73Pro)	LP	c.218G>C	p.(Arg73Pro)	LP	Tracewska 2021	RD	
00450776	067285	c.218G>C	p.(Arg73Pro)	LP	c.218G>C	p.(Arg73Pro)	LP	Hitti-Malin 2024	CD/CRD	
00450777	073880	c.218G>C	p.(Arg73Pro)	LP	c.218G>C	p.(Arg73Pro)	LP	Hitti-Malin 2024	CD/CRD	
n.a.	n.a.	c.218G>C	p.(Arg73Pro)	LP	c.218G>C	p.(Arg73Pro)	LP	LOVD data, personal communication		skeletal abnormalities + CRD

n.a.	n.a.	c.218G>C	p.(Arg73Pro)	LP	c.218G>C	p.(Arg73Pro)	LP	LOVD data, personal communication		Chiari malformation, dysproportioned small height growth, delayed motor development + LCA
n.a.	n.a.	c.218G>C	p.(Arg73Pro)	LP	c.218G>C	p.(Arg73Pro)	LP	LOVD data, personal communication	CRD	
n.a.	n.a.	c.218G>C	p.(Arg73Pro)	LP	c.218G>C	p.(Arg73Pro)	LP	LOVD data, personal communication	RCD	
n.a.	n.a.	c.218G>C	p.(Arg73Pro)	LP	c.218G>C	p.(Arg73Pro)	LP	LOVD data, personal communication		ADHD, low intelligence, autism + RCD
n.a.	n.a.	c.218G>C	p.(Arg73Pro)	LP	c.218G>C	p.(Arg73Pro)	LP	LOVD data, personal communication	RD	
00104990	A10.1	c.286G>A	p.(Glu96Lys)	LP	c.633_634del	p.(His211Glnfs*98)	P	de Castro-Miró 2016		SMDAX + CRD
00389789	1073	c.286G>A	p.(Glu96Lys)	LP	c.286G>A	p.(Glu96Lys)	LP	Weisschuh 2020	CRD	
00327459	FamPatII2	c.319T>C	p.(Tyr107His)	LP	c.319T>C	p.(Tyr107His)	LP	Kurashige 2019		Syndromic IRD (ALS, RCD, slowly progressive generalized muscle weakness, dysphagia)
00327460	FamPatII6 (sib)	c.319T>C	p.(Tyr107His)	LP	c.319T>C	p.(Tyr107His)	LP	Kurashige 2019		Syndromic IRD (childhood RCD, 50s-muscle weakness, 30s-Crohn’s disease)
00327508	Fam5	c.319T>C	p.(Tyr107His)	LP	c.347C>T	p.(Pro116Leu)	LP	Wang 2016		SMDAX + RCD
00327513	Fam1	c.319T>C	p.(Tyr107His)	LP	c.331G>A	p.(Val111Met)	LP	Suga 2016		Syndromic IRD (RCD, short stature)
00435041	n.a.	c.319T>C	p.(Tyr107His)	LP	c.347C>T	p.(Pro116Leu)	LP	n.a.	CRD	
00327514	Fam2	c.320A>G	p.(Tyr107Cys)	LP	c.320A>G	p.(Tyr107Cys)	LP	Suga 2016	RCD	
00387032	58	c.320A>G	p.(Tyr107Cys)	LP	c.320A>G	p.(Tyr107Cys)	LP	Jauregui 2020	RCD	
n.a.	n.a.	c.331G>A	p.(Val111Met)	LP	c.331G>A	p.(Val111Met)	LP	LOVD data, personal communication	RCD	
00389526	810	c.335_346del	p.(Leu112_ Leu115del)	LP	c.335_346del	p.(Leu112_ Leu115del)	LP	Weisschuh 2020	RCD	
n.a.	Patient 1	c.340_351dup	p.(Thr114_ Arg117dup)	VUS	c.340_351dup	p.(Thr114_ Arg117)dup	VUS	Chiu 2022		Syndromic IRD (CRD, macular staphyloma, and short stature)
00436590	2701426/49	c.347C>T	p.(Pro116Leu)	LP	c.115_117dup	p.(Met39dup)	VUS	Villafuerte-de la Cruz 2024	CRD	
00308988	n.a.	c.364G>A	p.(Asp122Asn)	LP	c.364G>A	p.(Asp122Asn)	LP	Sharon 2019	CRD	

00327504	Pat1	c.436_466del	p.(Glu146Serfs*6)	P	c.436_466del	p.(Glu146Serfs*6)	P	Khan 2015	eoRD(nyctalopia, RPE mottling, macular staphyloma)	
00327505	Pat2	c.436_466del	p.(Glu146Serfs*6)	P	c.436_466del	p.(Glu146Serfs*6)	P	Khan 2015	eoRD(nyctalopia, RPE mottling, macular staphyloma)	
n.a.	n.a.	c.545+1G>A	p.(Ala181Glnfs*6)	LP	c.545+1G>A	p.(Ala181Glnfs*6)	LP	Abu-Safieh-2013	CRD	
00327509	Fam6	c.545+1G>A	p.[Ser183*, Ala181Glnfs*6]	LP	c.545+1G>A	p.[Ser183*,Ala181Glnfs*6]	LP	Wang 2016		SMDAX + CRD
00248837	FamF1P1	c.545+1G>T	p.?	P	c.545+1G>T	p.?	P	Wheway 2015	CRD	
00327507	Fam1	c.643-23A>T	p.(=), p.?	LP	c.643-23A>T	p.(=), p.?	LP	Wang 2016		SMDAX + CRD
00327510	Fam7	c.643-23A>T	p.(=), p.?	LP	c.643-23A>T	p.(=), p.?	LP	Wang 2016		SMDAX + RCD
00331334	10DG1767, 10DG1768, 10DG1769	c.643-23A>T	p.(=), p.?	LP	c.643-23A>T	p.(=), p.?	LP	Maddirevula 2018		Skeletal dysplasia (short stature, hypermetropia, strabismus, rhizomelia, micrognathia)
00331336	13DG0374, 13DG0375	c.643-23A>T	p.(=), p.?	LP	c.643-23A>T	p.(=), p.?	LP	Maddirevula 2018		Skeletal dysplasia (short stature, narrow chest)
00363758	10DG1769	c.643-23A>T	p.(=), p.?	LP	c.643-23A>T	p.(=), p.?	LP	Patel 2016		JS (short stature) + CRD
00308987	n.a.	c.643-2A>G	p.?	P	c.643-2A>G	p.?	P	Sharon 2019	LCA	
00308986	n.a.	c.643-1G>C	p.?	P	c.643-1G>C	p.?	P	Sharon 2019	RCD	
n.a.	1_II-1	c.218G>C	p.(Arg73Pro)	LP	c.218G>C	p.(Arg73Pro)	LP	This study	CD	
n.a.	2_II-1	c.218G>C	p.(Arg73Pro)	LP	c.218G>C	p.(Arg73Pro)	LP	This study	CRD	
n.a.	3_II-1	c.73T>C	p.(Cys25Arg)	LP	c.373+91A>G	p.?	LP	This study	CRD	
n.a.	4_II-1	c.218G>C	p.(Arg73Pro)	LP	c.441_444del	p.(Glu148Alafs*13)	P	This study	CRD	
n.a.	5_II-1	c.19_21del	p.(Met7del)	VUS	c.291C>G	p.(Asn97Lys)	VUS	This study	CRD	
n.a.	6_II-1	c.96+1G>A	p.?	P	c.96+1G>A	p.?	P	This study	CRD	
n.a.	7_II-1	c.347C>T	p.(Pro116Leu)	LP	c.388_390del	p.(Glu130del)	LP	This study	CRD	
n.a.	8_II-1	c.218G>C	p.(Arg73Pro)	LP	c.286G>A	p.(Glu96Lys)	LP	This study	RCD	
n.a.	9_II-1	c.218G>C	p.(Arg73Pro)	LP	c.218G>C	p.(Arg73Pro)	LP	This study	RCD	
n.a.	10_II-1	c.218G>C	p.(Arg73Pro)	LP	c.218G>C	p.(Arg73Pro)	LP	This study	RCD	
n.a.	11_II-1	c.218G>C	p.(Arg73Pro)	LP	c.218G>C	p.(Arg73Pro)	LP	This study	RCD	
n.a.	12_II-1	c.218G>C	p.(Arg73Pro)	LP	c.218G>C	p.(Arg73Pro)	LP	This study	RCD	

n.a.	13_II-1	c.218G>C	p.(Arg73Pro)	LP	c.218G>C	p.(Arg73Pro)	LP	This study	RCD	
n.a.	14_II-1	c.218G>C	p.(Arg73Pro)	LP	c.218G>C	p.(Arg73Pro)	LP	This study		RCD + thoracic skeletal abnormalities
n.a.	15_II-1	c.143+3A>T	p.?	LP	c.143+3A>T	p.?	LP	This study	RCD	
n.a.	16_II-1	c.355C>T	p.Gln119*	P	c.355C>T	p.Gln119*	P	This study		eoRD + bilateral hip dysplasia, asymmetric bilateral hearing, early ovarian failure

Supplementary_Table_2: List of described syndromic and non-syndromic IRD patients carrying *CFAP410* biallelic variants. Data were retrieved from the LOVD database (www.LOVD.nl, accessed September 1st, 2024). ACMG, American College of Medical Genetics variant classification; ALS, amyotrophic lateral sclerosis; CD, cone dystrophy; CRD, cone-rod dystrophy; eoRD, early-onset Retinal degeneration; JS, Jeune syndrome; LP, likely pathogenic; n.a., not available; P, pathogenic; RCD, rod-cone dystrophy; RD, retinal degeneration (generic); SMDAX, Axial Spondylometaphyseal Dysplasia; VUS, variant of uncertain significance.

Reported patients	*CFAP410* _c.	*CFAP410* _p.	ACMG	Reference	Diagnosis
1	c.19_21del	p.(Met7del)	VUS	this study	CRD
2	c.26T>C	p.(Leu9Pro)	VUS	Shinbashi 2023	RCD
4	c.33_34ins AGCTGCACAGCGTGCA	p.(Ala12Serfs*60)	P	Carss 2017; Birtel 2018; Turro 2020; Tracewska 2021	CRD; RCD; RD
2	c.59_62del	p.(Val20Alafs*24)	P	Weisschuh 2024	RCD
1	c.73T>C	p.(Cys25Arg)	LP	this study	CRD
1	c.76T>C	p.(Trp26Arg)	P	Fadaie 2021	RCD
1	c.77+1G>C	p.(?)	P	LOVD data, personal communication	Skeletal dysplasia (short stature, narrow chest) + RCD
4	c.96+1G>A	p.(?)	P	Huang 2016; Rodriguez-Munoz 2020; this study	CRD
2	c.96+6T>A	p.(?)	LP	Wheway 2015; Weisschuh 2020	RCD; JS + CRD
1	c.96+670_97-41del	p.(?)	LP	Gustafson 2017	RCD
4	c.103del	p.(Ile35Phefs*10)	P	Abu-Safieh-2013; Wheway 2015; Maddirevula 2018	CRD; RCD; Skeletal Displasia + RD
1	c.115_117dup	p.(Met39dup)	VUS	Villafuerte-de la Cruz 2024	CRD
2	c.137C>T	p.(Thr46Met)	LP	Wang 2018; Liu-2020	RCD
1	c.143+3A>T	p.(?)	LP	this study	RCD
1	c.144-6_159del	p.(?)	P	Wang 2018	RCD
1	c.144_159del	p.(Ser48Argfs*6)	P	Liu-2020	RCD
1	c.182G>A	p.(Cys61Tyr)	P	Khan 2015	RD
1	c.209G>A	p.(Arg70Gln)	VUS	Hitti-Malin 2024	CRD
36	c.218G>C	p.(Arg73Pro)	LP	Wheway 2015; Wang 2016; Zhang 2016; Carss 2017; McInerney-Leo 2017; Lionel 2018; Holtan 2020; Rodriguez-Munoz 2020; Weisschuh 2020; Turro 2020; Fadaie 2021; Tracewska 2021; Weisschuh 2024; Hitti-Malin 2024; this study	CD; CRD; RCD; RD; SMDAX + RCD; JS + CRD; JS + RD; thoracic skeletal abnormalities + RCD; skeletal abnormalities + CRD; Chiari malformation, dysproportioned small height growth, delayed motor development + LCA; ADHD, low intelligence, autism + RCD
2	c.246C>A	p.(Tyr82*)	P	Rodriguez-Munoz 2020	CRD
1	c.269G>C	p.(Arg90Pro)	P	Carss 2017; Turro 2020	CRD
3	c.286G>A	p.(Glu96Lys)	LP	de Castro-Miró 2016; Weisschuh 2020; this study	CRD; RCD; SMDAX + CRD
1	c.291C>G	p.(Asn97Lys)	VUS	this study	CRD
1	c.293C>T	p.(Pro98Leu)	VUS	Weisschuh 2024	CRD
5	c.319T>C	p.(Tyr107His)	LP	Suga 2016; Wang 2016; Kurashige 2019	CRD; Syndromic IRD (ALS, RCD, slowly progressive generalized muscle weakness, dysphagia); Syndromic RD (childhood RCD, 50s-muscle weakness, 30s-Crohn’s disease); SMDAX + RCD; Syndromic RD (RCD, short stature); CRD

2	c.320A>G	p.(Tyr107Cys)	LP	Suga 2016; Jauregui 2020	RCD
2	c.331G>A	p.(Val111Met)	LP	LOVD data, personal communication; Suga 2016	RCD; Syndromic IRD (RCD, short stature)
1	c.335_346del	p.(Leu112_Leu115del)	LP	Weisschuh 2020	RCD
1	c.340_351dup	p.(Thr114_Arg117)dup	VUS	Chiu 2022	Syndromic IRD (CRD, macular staphyloma, and short stature)
4	c.347C>T	p.(Pro116Leu)	LP	Wang 2016; Villafuerte-de la Cruz 2024; this study	CRD; SMDAX
1	c.352_353ins ACCCTGCCGCGC	p.(Arg117_Leu118insHisProAl aAla)	LP	Huang 2016	CRD
1	c.355C>T	p.Gln119*	P	this study	eoRD + bilateral hip dysplasia, asymmetric bilateral hearing, early ovarian failure
1	c.364G>A	p.(Asp122Asn)	LP	Sharon 2019	CRD
1	c.364G>C	p.(Asp122His)	VUS	Zhang 2016	RCD
1	c.373+91A>G	p.(?)	LP	this study	CRD
1	c.374-3A>T	p.(?)	VUS	Birtel 2018	RD
1	c.388_390del	p.(Glu130del)	LP	this study	CRD
2	c.436_466del	p.(Glu146Serfs*6)	P	Khan 2015	eoRD (nyctalopia, RPE mottling, macular staphyloma)
1	c.441_444del	p.(Glu148Alafs*13)	P	this study	CRD
2	c.443_444del	p.(Glu148Glyfs*21)	P	Weisschuh 2024	RCD
1	c.480_481insT	p.(Leu161Serfs*9)	P	Wheway 2015	JS
1	c.482dup	p.(Ser162Glufs*8)	P	LOVD data, personal communication	CD
2	c.545+1G>A	p.[Ser183*,Ala181Glnfs*6]	LP	Abu-Safieh-2013; Wang 2016	CRD; SMDAX + CRD
1	c.545+1G>T	p.(?)	P	Wheway 2015	CRD
1	c.633_634del	p.(His211Glnfs*98)	P	de Castro-Miró 2016	SMDAX + CRD
5	c.643-23A>T	p.(=), p.(?)	LP	Wang 2016; Patel 2016; Maddirevula 2018	SMDAX + CRD; SMDAX + RCD; Skeletal dysplasia (short stature, hypermetropia, strabismus, rhizomelia, micrognathia); Skeletal dysplasia (short stature, narrow chest); JS (short stature, CRD)
1	c.643-2A>G	p.(?)	P	Sharon 2019	LCA
1	c.643-1G>C	p.(?)	P	Sharon 2019	RCD
3	c.671T>C	p.(Leu224Pro)	P	Wheway 2015	CRD; JS + CRD
2	c.673C>T	p.(Arg225Trp)	VUS	Kominami 2017	CD

Supplementary_Table_3: *CFAP410* variants detected in syndromic and non-syndromic IRD patients. Data were retrieved from the LOVD database (www.LOVD.nl, accessed September 1st, 2024) and combined with our case series. For most of the culprit variants detected in patients, the references of published studies are also provided. Variants lacking study references were found in unpublished patients undergone genetic testing. Variant c.673C>T was detected in one patients carrying biallelic *POC1B* pathogenic variants. ACMG, American College of Medical Genetics variant classification; ALS, amyotrophic lateral sclerosis; CD, cone dystrophy; CRD, cone-rod dystrophy; eoRD, early-onset retinal dystrophy; JS, Jeune syndrome; LCA, Leber Congenital Amaurosis; n.a., not available; RCD, rod-cone dystrophy; RD, retinal degeneration (generic); SMDAX, Axial Spondylometaphyseal Dysplasia; VUS, variant of uncertain significance.

Ensembl	Symbol	Alias	Disease Models	Description
ENSG00000198691	*ABCA4*		AR	ATP binding cassette subfamily A member 4
ENSG00000091262	*ABCC6*		AR;AD	ATP binding cassette subfamily C member 6
ENSG00000100997	*ABHD12*		AR	abhydrolase domain containing 12
ENSG00000107897	*ACBD5*		AR	acyl-CoA binding domain containing 5
ENSG00000100412	*ACO2*		AR	aconitase 2
ENSG00000168615	*ADAM9*		AR	ADAM metallopeptidase domain 9
ENSG00000140873	*ADAMTS18*		AR	ADAM metallopeptidase with thrombospondin type 1 motif 18
ENSG00000152990	*ADGRA3*	*GPR125*	AR	adhesion G protein-coupled receptor A3
ENSG00000164199	*ADGRV1*	*GPR98*	AR;AD	adhesion G protein-coupled receptor V1
ENSG00000141385	*AFG3L2*		AR;AD	AFG3 like matrix AAA peptidase subunit 2
ENSG00000135541	*AHI1*		AR	Abelson helper integration site 1
ENSG00000129221	*AIPL1*		AR	aryl hydrocarbon receptor interacting protein like 1
ENSG00000116127	*ALMS1*		AR	ALMS1 centrosome and basal body associated protein
ENSG00000169379	*ARL13B*		AR	ADP ribosylation factor like GTPase 13B
ENSG00000102931	*ARL2BP*		AR	ADP ribosylation factor like GTPase 2 binding protein
ENSG00000113966	*ARL6*		AR	ADP ribosylation factor like GTPase 6
ENSG00000162174	*ASRGL1*		AR	asparaginase like 1
ENSG00000118217	*ATF6*		AR	activating transcription factor 6
ENSG00000163635	*ATXN7*		AD	ataxin 7
ENSG00000148090	*AUH*		AR	AU RNA binding methylglutaconyl-CoA hydratase
ENSG00000214413	*BBIP1*		AR	BBSome interacting protein 1
ENSG00000174483	*BBS1*		AR	Bardet-Biedl syndrome 1
ENSG00000179941	*BBS10*		AR	Bardet-Biedl syndrome 10
ENSG00000181004	*BBS12*		AR	Bardet-Biedl syndrome 12
ENSG00000125124	*BBS2*		AR	Bardet-Biedl syndrome 2
ENSG00000140463	*BBS4*		AR	Bardet-Biedl syndrome 4
ENSG00000163093	*BBS5*		AR	Bardet-Biedl syndrome 5
ENSG00000138686	*BBS7*		AR	Bardet-Biedl syndrome 7
ENSG00000122507	*BBS9*		AR	Bardet-Biedl syndrome 9
ENSG00000167995	*BEST1*		AR;AD	bestrophin 1
ENSG00000130921	*C12orf65*		AR	chromosome 12 open reading frame 65
ENSG00000171155	*C1GALT1C1*		AR	C1GALT1 specific chaperone 1
ENSG00000223953	*C1QTNF5*		AD	C1q and TNF related 5
ENSG00000160226	*C21orf2*	*CFAP410*	AR	chromosome 21 open reading frame 2
ENSG00000179270	*C2orf71*		AR	chromosome 2 open reading frame 71
ENSG00000197603	*C5orf42*		AR	chromosome 5 open reading frame 42
ENSG00000156172	*C8orf37*		AR	chromosome 8 open reading frame 37
ENSG00000167434	*CA4*		AD	carbonic anhydrase 4
ENSG00000175544	*CABP4*		AR	calcium binding protein 4
ENSG00000102001	*CACNA1F*		XL	calcium voltage-gated channel subunit alpha1 F
ENSG00000151062	*CACNA2D4*		AR	calcium voltage-gated channel auxiliary subunit alpha2delta 4
ENSG00000149260	*CAPN5*		AD	calpain 5
ENSG00000048342	*CC2D2A*		AR	coiled-coil and C2 domain containing 2A
ENSG00000107736	*CDH23*		AR	cadherin related 23
ENSG00000062038	*CDH3*		AR	cadherin 3
ENSG00000148600	*CDHR1*		AR	cadherin related family member 1
ENSG00000110274	*CEP164*		AR	centrosomal protein 164
ENSG00000198707	*CEP290*		AR	centrosomal protein 290
ENSG00000106477	*CEP41*		AR	centrosomal protein 41
ENSG00000148019	*CEP78*		AR	centrosomal protein 78

ENSG00000173588	*CEP83*	*CCDC41*	AR	centrosomal protein 83
ENSG00000188452	*CERKL*		AR	ceramide kinase like
ENSG00000188419	*CHM*		XL-Dom	CHM Rab escort protein 1
ENSG00000128656	*CHN1*		AD	chimerin 1
ENSG00000136425	*CIB2*		AR	calcium and integrin binding family member 2
ENSG00000145354	*CISD2*		AR	CDGSH iron sulfur domain 2
ENSG00000188603	*CLN3*		AR	CLN3 battenin
ENSG00000102805	*CLN5*		AR	CLN5 intracellular trafficking protein
ENSG00000128973	*CLN6*		AR	CLN6 transmembrane ER protein
ENSG00000182372	*CLN8*		AR	CLN8 transmembrane ER and ERGIC protein
ENSG00000163646	*CLRN1*		AR	clarin 1
ENSG00000198515	*CNGA1*		AR	cyclic nucleotide gated channel alpha 1
ENSG00000144191	*CNGA3*		AR	cyclic nucleotide gated channel alpha 3
ENSG00000070729	*CNGB1*		AR	cyclic nucleotide gated channel beta 1
ENSG00000170289	*CNGB3*		AR	cyclic nucleotide gated channel beta 3
ENSG00000158158	*CNNM4*		AR	cyclin and CBS domain divalent metal cation transport mediator 4
ENSG00000060718	*COL11A1*		AD	collagen type XI alpha 1 chain
ENSG00000139219	*COL2A1*		AD	collagen type II alpha 1 chain
ENSG00000112280	*COL9A1*		AR;AD	collagen type IX alpha 1 chain
ENSG00000134376	*CRB1*		AR	crumbs 1 cell polarity complex component
ENSG00000105392	*CRX*		AR;AD	cone-rod homeobox
ENSG00000104218	*CSPP1*		AR	centrosome and spindle pole associated protein 1
ENSG00000138061	*CYP1B1*		AR	cytochrome P450 family 1 subfamily B member 1
ENSG00000145476	*CYP4V2*		AR	cytochrome P450 family 4 subfamily V member 2
ENSG00000117682	*DHDDS*		AR	dehydrodolichyl diphosphate synthase subunit
ENSG00000140829	*DHX38*		AR	DEAH-box helicase 38
ENSG00000156171	*DRAM2*		AR	DNA damage regulated autophagy modulator 2
ENSG00000197057	*DTHD1*		AR	death domain containing 1
ENSG00000115380	*EFEMP1*		AD	EGF containing fibulin like extracellular matrix protein 1
ENSG00000118402	*ELOVL4*		AR;AD	ELOVL fatty acid elongase 4
ENSG00000127463	*EMC1*		AR	ER membrane protein complex subunit 1
ENSG00000225830	*ERCC6*		AR	ERCC excision repair 6 chromatin remodeling factor
ENSG00000188107	*EYS*		AR	eyes shut homolog (Drosophila)
ENSG00000170264	*FAM161A*		AR	family with sequence similarity 161 member A
ENSG00000163013	*FBXO41*		AR	F-box protein 41
ENSG00000162769	*FLVCR1*		AR	feline leukemia virus subgroup C cellular receptor 1
ENSG00000054598	*FOXC1*		AD	forkhead box C1
ENSG00000137273	*FOXF2*		AD	forkhead box F2
ENSG00000186765	*FSCN2*		AD	fascin actin-bundling protein 2 retinal
ENSG00000174804	*FZD4*		AD	frizzled class receptor 4
ENSG00000109586	*GALNT7*		AR	polypeptide N-acetylgalactosaminyltransferase 7
ENSG00000156466	*GDF6*		AR;AD	growth differentiation factor 6
ENSG00000114349	*GNAT1*		AR;AD	G protein subunit alpha transducin 1
ENSG00000134183	*GNAT2*		AR	G protein subunit alpha transducin 2
ENSG00000078369	*GNB1*		AR	G protein subunit beta 1
ENSG00000111664	*GNB3*		AR	G protein subunit beta 3
ENSG00000090581	*GNPTG*		AR	N-acetylglucosamine-1-phosphate transferase gamma subunit
ENSG00000101850	*GPR143*		XL	G protein-coupled receptor 143
ENSG00000188888	*GPR179*		AR	G protein-coupled receptor 179
ENSG00000185974	*GRK1*		AR	G protein-coupled receptor kinase 1
ENSG00000113262	*GRM6*		AR	glutamate metabotropic receptor 6
ENSG00000030582	*GRN*		AR;AD	granulin precursor

ENSG00000048545	*GUCA1A*		AD	guanylate cyclase activator 1A
ENSG00000112599	*GUCA1B*		AD	guanylate cyclase activator 1B
ENSG00000132518	*GUCY2D*		AR;AD	guanylate cyclase 2D retinal
ENSG00000170445	*HARS*		AR	histidyl-tRNA synthetase
ENSG00000112855	*HARS2*		AR	histidyl-tRNA synthetase 2 mitochondrial
ENSG00000068024	*HDAC4*		AR	histone deacetylase 4
ENSG00000215612	*HMX1*		AR	H6 family homeobox 1
ENSG00000105991	*HOXA1*		AR	homeobox A1
ENSG00000120094	*HOXB1*		AR	homeobox B1
ENSG00000101365	*IDH3B*		AR	isocitrate dehydrogenase 3 (NAD(+)) beta
ENSG00000163913	*IFT122*		AR	intraflagellar transport 122
ENSG00000187535	*IFT140*		AR	intraflagellar transport 140
ENSG00000138002	*IFT172*		AR	intraflagellar transport 172
ENSG00000100360	*IFT27*		AR	intraflagellar transport 27
ENSG00000119650	*IFT43*		AR	intraflagellar transport 43
ENSG00000068885	*IFT80*		AR	intraflagellar transport 80
ENSG00000032742	*IFT88*		AR	intraflagellar transport 88
ENSG00000163395	*IGFN1*		AR	immunoglobulin-like and fibronectin type III domain containing 1
ENSG00000073009	*IKBKG*		XL	inhibitor of nuclear factor kappa B kinase subunit gamma
ENSG00000106348	*IMPDH1*		AD	inosine monophosphate dehydrogenase 1
ENSG00000112706	*IMPG1*		AR;AD	interphotoreceptor matrix proteoglycan 1
ENSG00000081148	*IMPG2*		AR	interphotoreceptor matrix proteoglycan 2
ENSG00000148384	*INPP5E*		AR	inositol polyphosphate-5-phosphatase E
ENSG00000119509	*INVS*		AR	inversin
ENSG00000173226	*IQCB1*		AR	IQ motif containing B1
ENSG00000136156	*ITM2B*		AD	integral membrane protein 2B
ENSG00000101384	*JAG1*		AD	jagged canonical Notch ligand 1
ENSG00000115474	*KCNJ13*		AR;AD	potassium voltage-gated channel subfamily J member 13
ENSG00000168263	*KCNV2*		AR	potassium voltage-gated channel modifier subfamily V member 2
ENSG00000243335	*KCTD7*		AR	potassium channel tetramerization domain containing 7
ENSG00000122778	*KIAA1549*		AR	KIAA1549
ENSG00000138160	*KIF11*		AD	kinesin family member 11
ENSG00000139116	*KIF21A*		AD	kinesin family member 21A
ENSG00000088970	*KIZ*	*PLK1S1*	AR	kizuna centrosomal protein
ENSG00000122550	*KLHL7*		AD	kelch like family member 7
ENSG00000135338	*LCA5*		AR	LCA5 lebercilin
ENSG00000121207	*LRAT*		AR	lecithin retinol acyltransferase
ENSG00000183423	*LRIT3*		AR	leucine rich repeat Ig-like and transmembrane domains 3
ENSG00000162337	*LRP5*		AR;AD	LDL receptor related protein 5
ENSG00000119681	*LTBP2*		AR	latent transforming growth factor beta binding protein 2
ENSG00000163818	*LZTFL1*		AR	leucine zipper transcription factor like 1
ENSG00000111837	*MAK*		AR	male germ cell associated kinase
ENSG00000114738	*MAPKAPK3*		AR	mitogen-activated protein kinase-activated protein kinase 3
ENSG00000153208	*MERTK*		AR	MER proto-oncogene tyrosine kinase
ENSG00000116688	*MFN2*		AD	mitofusin 2
ENSG00000235718	*MFRP*		AR	membrane frizzled-related protein
ENSG00000164073	*MFSD8*		AR	major facilitator superfamily domain containing 8
ENSG00000125863	*MKKS*		AR	McKusick-Kaufman syndrome
ENSG00000011143	*MKS1*		AR	Meckel syndrome type 1
ENSG00000198899	*MT-ATP6*		MT	mitochondrially encoded ATP synthase 6
ENSG00000228253	*MT-ATP8*		MT	mitochondrially encoded ATP synthase 8
ENSG00000198804	*MT-CO1*		MT	mitochondrially encoded cytochrome c oxidase I

ENSG00000198712	*MT-CO2*	MT	mitochondrially encoded cytochrome c oxidase II
ENSG00000198938	*MT-CO3*	MT	mitochondrially encoded cytochrome c oxidase III
ENSG00000198727	*MT-CYB*	MT	mitochondrially encoded cytochrome b
ENSG00000198888	*MT-ND1*	MT	mitochondrially encoded NADH:ubiquinone oxidoreductase core subunit 1
ENSG00000198763	*MT-ND2*	MT	mitochondrially encoded NADH:ubiquinone oxidoreductase core subunit 2
ENSG00000198840	*MT-ND3*	MT	mitochondrially encoded NADH:ubiquinone oxidoreductase core subunit 3
ENSG00000198886	*MT-ND4*	MT	mitochondrially encoded NADH:ubiquinone oxidoreductase core subunit 4
ENSG00000212907	*MT-ND4L*	MT	mitochondrially encoded NADH:ubiquinone oxidoreductase core subunit 4L
ENSG00000198786	*MT-ND5*	MT	mitochondrially encoded NADH:ubiquinone oxidoreductase core subunit 5
ENSG00000198695	*MT-ND6*	MT	mitochondrially encoded NADH:ubiquinone oxidoreductase core subunit 6
ENSG00000210176	*MT-TH*	MT	mitochondrially encoded tRNA histidine
ENSG00000209082	*MT-TL1*	MT	mitochondrially encoded tRNA leucine 1 (UUA/G)
ENSG00000210196	*MT-TP*	MT	mitochondrially encoded tRNA proline
ENSG00000210184	*MT-TS2*	MT	mitochondrially encoded tRNA serine 2 (AGU/C)
ENSG00000107951	*MTPAP*	AR	mitochondrial poly(A) polymerase
ENSG00000138823	*MTTP*	AR	microsomal triglyceride transfer protein
ENSG00000110921	*MVK*	AR	mevalonate kinase
ENSG00000137474	*MYO7A*	AR	myosin VIIA
ENSG00000034971	*MYOC*	AD	myocilin
ENSG00000124479	*NDP*	XL	NDP norrin cystine knot growth factor
ENSG00000023228	*NDUFS1*	AR	NADH:ubiquinone oxidoreductase core subunit S1
ENSG00000117650	*NEK2*	AR	NIMA related kinase 2
ENSG00000162992	*NEUROD1*	AR	neuronal differentiation 1
ENSG00000116044	*NFE2L2*	AR	nuclear factor erythroid 2 like 2
ENSG00000173614	*NMNAT1*	AR	nicotinamide nucleotide adenylyltransferase 1
ENSG00000144061	*NPHP1*	AR	nephrocystin 1
ENSG00000113971	*NPHP3*	AR	nephrocystin 3
ENSG00000131697	*NPHP4*	AR	nephrocystin 4
ENSG00000126368	*NR1D1*	AR	nuclear receptor subfamily 1 group D member 1
ENSG00000031544	*NR2E3*	AR;AD	nuclear receptor subfamily 2 group E member 3
ENSG00000175745	*NR2F1*	AR	nuclear receptor subfamily 2 group F member 1
ENSG00000129535	*NRL*	AR;AD	neural retina leucine zipper
ENSG00000171773	*NXNL1*	AR	nucleoredoxin like 1
ENSG00000188937	*NYX*	XL-Rec	nyctalopin
ENSG00000065154	*OAT*	AR	ornithine aminotransferase
ENSG00000104044	*OCA2*	AR	OCA2 melanosomal transmembrane protein
ENSG00000046651	*OFD1*	XL	OFD1 centriole and centriolar satellite protein
ENSG00000198836	*OPA1*	AD	OPA1 mitochondrial dynamin like GTPase
ENSG00000125741	*OPA3*	AR;AD	OPA3 outer mitochondrial membrane lipid metabolism regulator
ENSG00000102076	*OPN1LW*	XL	opsin 1 long wave sensitive
ENSG00000147380	*OPN1MW*	XL	opsin 1 medium wave sensitive
ENSG00000128617	*OPN1SW*	AD	opsin 1 short wave sensitive
ENSG00000123240	*OPTN*	AD	optineurin
ENSG00000165588	*OTX2*	AR;AD	orthodenticle homeobox 2
ENSG00000125779	*PANK2*	AR	pantothenate kinase 2
ENSG00000075891	*PAX2*	AD	paired box 2
ENSG00000007372	*PAX6*	AD	paired box 6
ENSG00000150275	*PCDH15*	AR	protocadherin related 15
ENSG00000161217	*PCYT1A*	AR	phosphate cytidylyltransferase 1 choline alpha
ENSG00000178104	*PDE4DIP*	AR	phosphodiesterase 4D interacting protein
ENSG00000132915	*PDE6A*	AR	phosphodiesterase 6A
ENSG00000133256	*PDE6B*	AR;AD	phosphodiesterase 6B

ENSG00000095464	*PDE6C*	AR	phosphodiesterase 6C
ENSG00000156973	*PDE6D*	AR	phosphodiesterase 6D
ENSG00000185527	*PDE6G*	AR	phosphodiesterase 6G
ENSG00000139053	*PDE6H*	AR	phosphodiesterase 6H
ENSG00000186862	*PDZD7*	AR	PDZ domain containing 7
ENSG00000121440	*PDZRN3*	AR	PDZ domain containing ring finger 3
ENSG00000127980	*PEX1*	AR	peroxisomal biogenesis factor 1
ENSG00000157911	*PEX10*	AR	peroxisomal biogenesis factor 10
ENSG00000142655	*PEX14*	AR	peroxisomal biogenesis factor 14
ENSG00000121680	*PEX16*	AR	peroxisomal biogenesis factor 16
ENSG00000162735	*PEX19*	AR	peroxisomal biogenesis factor 19
ENSG00000164751	*PEX2*	AR	peroxisomal biogenesis factor 2
ENSG00000139197	*PEX5*	AR	peroxisomal biogenesis factor 5
ENSG00000124587	*PEX6*	AR	peroxisomal biogenesis factor 6
ENSG00000112357	*PEX7*	AR	peroxisomal biogenesis factor 7
ENSG00000165462	*PHOX2A*	AR	paired like homeobox 2a
ENSG00000107537	*PHYH*	AR	phytanoyl-CoA 2-hydroxylase
ENSG00000091622	*PITPNM3*	AD	PITPNM family member 3
ENSG00000164093	*PITX2*	AD	paired like homeodomain 2
ENSG00000127472	*PLA2G5*	AR	phospholipase A2 group V
ENSG00000032444	*PNPLA6*	AR	patatin like phospholipase domain containing 6
ENSG00000139323	*POC1B*	AR	POC1 centriolar protein B
ENSG00000140521	*POLG*	AR;AD	DNA polymerase gamma catalytic subunit
ENSG00000086717	*PPEF1*	AR	protein phosphatase with EF-hand domain 1
ENSG00000131238	*PPT1*	AR	palmitoyl-protein thioesterase 1
ENSG00000214140	*PRCD*	AR	photoreceptor disc component
ENSG00000112238	*PRDM13*	AR	PR/SET domain 13
ENSG00000007062	*PROM1*	AR;AD	prominin 1
ENSG00000117360	*PRPF3*	AD	pre-mRNA processing factor 3
ENSG00000105618	*PRPF31*	AD	pre-mRNA processing factor 31
ENSG00000136875	*PRPF4*	AD	pre-mRNA processing factor 4
ENSG00000101161	*PRPF6*	AD	pre-mRNA processing factor 6
ENSG00000174231	*PRPF8*	AD	pre-mRNA processing factor 8
ENSG00000112619	*PRPH2*	AR;AD	peripherin 2
ENSG00000157869	*RAB28*	AR	RAB28 member RAS oncogene family
ENSG00000182185	*RAD51B*	AR	RAD51 paralog B
ENSG00000173976	*RAX2*	AD	retina and anterior neural fold homeobox 2
ENSG00000107618	*RBP3*	AR	retinol binding protein 3
ENSG00000138207	*RBP4*	AR	retinol binding protein 4
ENSG00000136144	*RCBTB1*	AR	RCC1 and BTB domain containing protein 1
ENSG00000198570	*RD3*	AR	retinal degeneration 3
ENSG00000139988	*RDH12*	AR;AD	retinol dehydrogenase 12 (all-trans/9-cis/11-cis)
ENSG00000135437	*RDH5*	AR	retinol dehydrogenase 5
ENSG00000115255	*REEP6*	AR	receptor accessory protein 6
ENSG00000148604	*RGR*	AR;AD	retinal G protein coupled receptor
ENSG00000108370	*RGS9*	AR	regulator of G protein signaling 9
ENSG00000186326	*RGS9BP*	AR	regulator of G protein signaling 9 binding protein
ENSG00000163914	*RHO*	AR;AD	rhodopsin
ENSG00000079841	*RIMS1*	AD	regulating synaptic membrane exocytosis 1
ENSG00000140522	*RLBP1*	AR	retinaldehyde binding protein 1
ENSG00000154134	*ROBO3*	AR	roundabout guidance receptor 3
ENSG00000149489	*ROM1*	AD	retinal outer segment membrane protein 1

ENSG00000104237	*RP1*		AR;AD	RP1 axonemal microtubule associated
ENSG00000183638	*RP1L1*			RP1 like 1
ENSG00000102218	*RP2*		XL	RP2 ARL3 GTPase activating protein
ENSG00000164610	*RP9*		AD	RP9 pre-mRNA splicing factor
ENSG00000116745	*RPE65*		AR;AD	RPE65 retinoid isomerohydrolase
ENSG00000156313	*RPGR*		XL	retinitis pigmentosa GTPase regulator
ENSG00000092200	*RPGRIP1*		AR	retinitis pigmentosa GTPase regulator interacting protein 1
ENSG00000103494	*RPGRIP1L*		AR	RPGRIP1 like
ENSG00000114391	*RPL24*		AR	ribosomal protein L24
ENSG00000102104	*RS1*		XL-Dom	retinoschisin 1
ENSG00000130561	*SAG*		AR	S-antigen visual arrestin
ENSG00000101115	*SALL4*		AD	spalt like transcription factor 4
ENSG00000111961	*SASH1*		AR	SAM and SH3 domain containing 1
ENSG00000054282	*SDCCAG8*		AR	serologically defined colon cancer antigen 8
ENSG00000196189	*SEMA4A*		AR	semaphorin 4A
ENSG00000074621	*SLC24A1*		AR	solute carrier family 24 member 1
ENSG00000164209	*SLC25A46*		AR	solute carrier family 25 member 46
ENSG00000164175	*SLC45A2*		AR	solute carrier family 45 member 2
ENSG00000188687	*SLC4A5*		AR;AD	solute carrier family 4 member 5
ENSG00000013293	*SLC7A14*		AR	solute carrier family 7 member 14
ENSG00000080503	*SMARCA2*		AR	SWI/SNF related matrix associated actin dependent regulator of chromatin subfamily a member 2
ENSG00000144028	*SNRNP200*		AR;AD	small nuclear ribonucleoprotein U5 subunit 200
ENSG00000042317	*SPATA7*		AR	spermatogenesis associated 7
ENSG00000197912	*SPG7*		AR;AD	SPG7 paraplegin matrix AAA peptidase subunit
ENSG00000072080	*SPP2*		AR	secreted phosphoprotein 2
ENSG00000128039	*SRD5A3*		AR	steroid 5 alpha-reductase 3
ENSG00000184895	*SRY*			sex determining region Y
ENSG00000187079	*TEAD1*		AD	TEA domain transcription factor 1
ENSG00000120156	*TEK*		AD	TEK receptor tyrosine kinase
ENSG00000126953	*TIMM8A*		XL	translocase of inner mitochondrial membrane 8A
ENSG00000100234	*TIMP3*		AD	TIMP metallopeptidase inhibitor 3
ENSG00000171202	*TMEM126A*		AR	transmembrane protein 126A
ENSG00000205084	*TMEM231*		AR	transmembrane protein 231
ENSG00000155755	*TMEM237*		AR	transmembrane protein 237
ENSG00000164953	*TMEM67*		AR	transmembrane protein 67
ENSG00000197579	*TOPORS*		AD	TOP1 binding arginine/serine rich protein
ENSG00000166340	*TPP1*	*TPPA*	AR	tripeptidyl peptidase 1
ENSG00000213689	*TREX1*		AD	three prime repair exonuclease 1
ENSG00000119401	*TRIM32*		AR	tripartite motif containing 32
ENSG00000153827	*TRIP12*		AR	thyroid hormone receptor interactor 12
ENSG00000134160	*TRPM1*		AR	transient receptor potential cation channel subfamily M member 1
ENSG00000106025	*TSPAN12*		AD	tetraspanin 12
ENSG00000123607	*TTC21B*		AR	tetratricopeptide repeat domain 21B
ENSG00000100154	*TTC28*		AR	tetratricopeptide repeat domain 28
ENSG00000165533	*TTC8*		AR	tetratricopeptide repeat domain 8
ENSG00000119685	*TTLL5*		AR	tubulin tyrosine ligase like 5
ENSG00000137561	*TTPA*		AR;AD	alpha tocopherol transfer protein
ENSG00000166402	*TUB*		AR	tubby bipartite transcription factor
ENSG00000258947	*TUBB3*		AD	tubulin beta 3 class III
ENSG00000137822	*TUBGCP4*		AR	tubulin gamma complex associated protein 4
ENSG00000112041	*TULP1*		AR	tubby like protein 1

ENSG00000077498	*TYR*		AR	tyrosinase
ENSG00000107165	*TYRP1*		AR	tyrosinase related protein 1
ENSG00000109103	*UNC119*		AD	unc-119 lipid binding chaperone
ENSG00000006611	*USH1C*		AR	USH1 protein network component harmonin
ENSG00000182040	*USH1G*		AR	USH1 protein network component sans
ENSG00000042781	*USH2A*		AR	usherin
ENSG00000038427	*VCAN*		AD	versican
ENSG00000132549	*VPS13B*		AR	vacuolar protein sorting 13 homolog B
ENSG00000143951	*WDPCP*		AR	WD repeat containing planar cell polarity effector
ENSG00000157796	*WDR19*		AR	WD repeat domain 19
ENSG00000119333	*WDR34*		AR	WD repeat domain 34
ENSG00000118965	*WDR35*		AR	WD repeat domain 35
ENSG00000109501	*WFS1*		AR;AD	wolframin ER transmembrane glycoprotein
ENSG00000095397	*WHRN*	*DFNB31*	AR	whirlin
ENSG00000056277	*ZNF280C*		AR	zinc finger protein 280C
ENSG00000175213	*ZNF408*		AR	zinc finger protein 408
ENSG00000102935	*ZNF423*		AR;AD	zinc finger protein 423 (AD also mentioned in OMIM for Joubert…)
ENSG00000163795	*ZNF513*		AR	zinc finger protein 513

Supplementary_Table_4: List of genes contained in GEDi (v6).

Primer name	Primer Sequence	Technique
EG117_*CFAP410* _Rev	TCACGTTGCCTCCTCCTCG	TA cloning
EG118_*CFAP410* _Forw	ACTGTGCTGGTCTCCGTGC	TA cloning
CFAP410 _EX1_F	GCATGAGCAGGAGCAGAG	Sanger Sequencing
CFAP410 _EX1_R	CTCTCCTATTCGACTTGGTCC	Sanger Sequencing
CFAP410 _EX3_F	GTGGCTTCGACAGTTCTCTG	Sanger Sequencing
CFAP410 _EX3_R	GTTATGTCACAGTGCTGGGTC	Sanger Sequencing
CFAP410 _EX4_F	GGACACAGGTCCCATGAGTG	Sanger Sequencing
CFAP410 _EX4_R	GACTTCACAGGACACATCTATGTCC	Sanger Sequencing
CFAP410 _EX5_F	GGAGTTGTCCCTGGAGGTG	Sanger Sequencing
CFAP410 _EX5_R	CTGTCCCTCACTTCCCAGAG	Sanger Sequencing
CFAP410 _EX4_F_RTPCR	GTCAACAGCATCTCCACCC	RT-PCR
CFAP410 _EX5_R_RTPCR	GCTGAGGGAGCTCAGTGTG	RT-PCR

Supplementary_Table_5: *CFAP410* primers used in this study
